# Exon-6 truncated p53 Suppresses Innate Immune Responses to Facilitate Immune Evasion in Bladder Cancer

**DOI:** 10.7150/ijbs.131491

**Published:** 2026-09-11

**Authors:** Chi Zhang, Wenjie Wei, Senming Cao, Zhi Li, Chuang Wang, Wen Tao, Yuhao Dong, Chunyu Liu, Shun Wu, Shuo Tian, Qingbo Huang, Baojun Wang, Hongzhao Li, Xin Ma, Xu Zhang, Yan Huang

**Affiliations:** 1 Senior Department of Urology, Chinese PLA General Hospital, Beijing, China.; 2 School of medicine, Nankai University, Tianjin, 300071, China.; 3 Department of Urology Laboratory, Chinese PLA General Hospital, Beijing, China.; 4 Medical School of PLA, Beijing, China.; 5 Department of Urology, The Affiliated Hospital of Qingdao University, Qingdao, 266000, China.

**Keywords:** truncated p53, type I interferon, innate immune, immune evasion, bladder cancer immunotherapy response

## Abstract

Previous studies have reported that truncated p53 exhibits pro-tumorigenic properties; however, whether it contributes to immune evasion by influencing the immune microenvironment remains unclear. Here, we report that exon-6-truncated p53 isoforms (E6-trunc p53) inhibit type I interferon (IFN-I) signaling, thereby supporting tumor cell survival and enabling immune evasion. Ectopic expression of E6-trunc p53 reduces T-cell infiltration into tumors and markedly promotes *in vivo* tumor growth in an immune-dependent manner. Mechanistically, E6-trunc p53 interacts with TBK1, thereby preventing cell-intrinsic activation of innate immune pathways and suppressing IFN-I-mediated antitumor immunity. Additionally, silencing E6-trunc p53 leads to robust upregulation of IFN-I signaling and induces tumor cell death. Deletion of TBK1 or IFN-β markedly rescues the cell death triggered by knockdown of E6-trunc p53. Notably, TP53 truncating mutations correlate inversely with the extent of T-cell infiltration and predict poor responsiveness to immunotherapy in patients with bladder cancer. Collectively, our findings reveal a novel oncogenic role of E6-trunc p53 in modulating IFN-I signaling and driving tumor malignancy and immune evasion.

## Introduction

Bladder cancer is one of the most common malignancies of the urinary system and ranks as the ninth most frequently diagnosed cancer worldwide 1. It is classified into non-muscle-invasive (NMIBC; comprising ~75%) and muscle-invasive (MIBC; representing ~25%) types according to the depth of tumor invasion 2. MIBC carries a high risk of distant metastasis, is associated with poor survival, and poses a significant therapeutic challenge 2. For eligible patients with MIBC, cisplatin-based neoadjuvant chemotherapy followed by radical cystectomy and pelvic lymph node dissection represents the standard treatment; for patients with radiographic pelvic lymph node involvement (cN+), neoadjuvant chemotherapy may also be considered to facilitate tumor downstaging and conversion to resectability 3. In recent years, immunotherapy with PD-1/PD-L1 inhibitors has transformed the clinical management of bladder cancer, improved pathological complete response and enabled strategies for bladder preservation 4. Nonetheless, many patients continue to experience suboptimal clinical outcomes.

Emerging evidence supports two distinct, and thought to be mutually exclusive, genetic pathways in the pathogenesis of urothelial bladder cancer, driven respectively by FGFR3 or TP53 mutations 5-7. Unlike FGFR3, which shows the highest mutation frequency in NMIBC, TP53 mutations represent the most prevalent genetic alteration in MIBC 8. The mutational patterns of TP53 in cancer, including bladder cancer, can be broadly classified into two main types: missense mutations and truncation mutations (nonsense mutations, frameshift variants, splice-site variants, and in-frame deletions) 9, 10. TP53 missense mutations typically disrupt the tumor-suppressive function of p53 while simultaneously conferring novel gain-of-function properties that promote carcinogenesis 11, 12. By contrast, TP53 truncation mutations are generally regarded as producing shortened, nonfunctional p53 proteins, thereby eliminating the gene's tumor-suppressive activity 10, 12.

Type I interferon (IFN-I) signaling, which drives the production of cytokines and chemokines, plays a critical role in regulating antitumor T-cell responses and modulating resistance to immunotherapy 13. The production of IFN-I is primarily governed by the activation of pattern recognition receptors (PRRs) in the innate immune system 14, 15. Notably, the cGAS-STING signaling axis plays an especially important role in the tumor microenvironment by recognizing cytoplasmic pools of free double-stranded DNA (dsDNA), a phenomenon driven by underlying genomic instability 16. Tumor cells frequently adopt diverse strategies to suppress IFN-I signaling, thereby facilitating immune evasion 17, 18. Previous studies have shown that mutant p53 suppresses the cGAS-STING-IFN-I signaling axis to promote tumor growth via immune escape 19, but whether E6-trunc p53 contributes to the regulation of PRR-mediated antitumor immunity remains unexplored.

In this study, we report that TP53 exon-6 truncating mutations occur frequently in bladder cancer, and the resulting E6-trunc p53 markedly suppresses innate immune signaling and promotes immune evasion by binding to TBK1. Moreover, inhibition of E6-trunc p53 induces a robust activation of IFN-I signaling and triggers interferon-dependent cell death. Ectopic expression of E6-trunc p53 variants reduces T-cell infiltration and impairs the efficacy of PD-1 blockade. Clinically, TP53 truncating mutations are associated with poor prognosis and predict resistance to immunotherapy in patients with bladder cancer. Thus, our findings suggest that E6-trunc p53 drives tumor malignancy and immune evasion by acquiring novel pro-oncogenic activities, rather than merely through loss of function.

## Results

### E6-trunc p53 drives tumor progression via immunosuppression

Previous studies have shown that truncating mutations in TP53 are associated with poor prognosis in multiple cancer types 20-24. Analysis of bladder cancer datasets revealed that truncating mutations represent the second most frequent class after missense mutations, accounting for 24.35%, with p53Q192* and p53R213* in exon 6 being the most prevalent (Figure 1A; Figure S1 A-B). Survival analysis confirmed that patients harboring truncating TP53 mutations had worse outcomes compared to those with wild-type TP53 (Figure S1C). To investigate the biological functions of E6-trunc p53, we expressed p53Q192* and p53R213* in the human bladder cancer cell line SW780, and the corresponding murine variants p53Q189* and p53R210* in MB49 cells (Figure S1D). Immunofluorescence demonstrated that E6-trunc p53 localized predominantly to the cytoplasm, in contrast to the nuclear distribution of wild-type p53 (Figure S1E). Cell proliferation and colony formation assays revealed that E6-trunc p53 had no significant effect on the *in vitro* growth rates of bladder cancer cells (Figure 1B; Figure S1F).

To assess the effect of E6-trunc p53 on tumor growth *in vivo*, E6-trunc p53-expressing MB49 cells were injected subcutaneously into immunocompromised mice. Our results showed that the E6-trunc p53 had no significant impact on tumor growth in BALB/c nude mice (Figure 1C). By contrast, syngeneic transplantation into immunocompetent C57BL/6J mice revealed that E6-trunc p53 markedly enhanced tumor progression (Figure 1D). Consistently, in orthotopic bladder tumor models, bioluminescence imaging and histological analysis demonstrated accelerated growth of E6-trunc p53 tumors compared with controls (Figure 1 E-F). To further validate p53 expression in the xenograft tumors, we performed p53 immunohistochemical staining on tumor sections from both control and E6-trunc p53-overexpressing mice. The staining pattern was consistent with the immunofluorescence results, showing predominant cytoplasmic localization of E6-trunc p53 in the tumor tissues (Figure S2C). Together, these findings indicate that E6-trunc p53 confers an immune-dependent gain-of-function phenotype that promotes bladder tumor growth *in vivo* only in hosts with an intact immune system.

### Ectopic expression of E6-trunc p53 variants potently restricts T cell infiltration

To define how E6-trunc p53 shapes antitumor immunity, we quantified immune effector cells in control and E6-trunc p53 tumors by flow cytometry. The gating strategy for analyzing changes in immune effector cell subsets within murine transplanted tumors is shown in Figure S2A. In MB49 tumors, expression of E6-trunc p53 markedly reduced infiltration of CD8+ T cells and CD4+ T cells, while differences in NK cells were not statistically significant (Figure 2A). Immunohistochemistry and immunofluorescence corroborated these observations, revealing reduced infiltration of CD8+ cytotoxic T cells and CD4+ helper T cells, as well as impaired expression of the cytotoxic marker granzyme B (GZMB), in both subcutaneous and orthotopic E6-trunc p53-expressing bladder tumors (Figure 2B-D). To determine whether E6-trunc p53 affects macrophage polarization in the tumor microenvironment, we performed double immunofluorescence staining for F4/80 and CD206 in xenograft tumor tissues. The E6-trunc p53 group showed a significantly increased proportion of CD206-positive cells among F4/80-positive macrophages compared with the control group, suggesting enhanced polarization toward an M2-like phenotype (Figure 2E). Because CD4+ T cells can support B-cell-mediated antitumor immunity, we further examined B-cell infiltration by CD19 immunofluorescence staining in tumor sections. E6-trunc p53-expressing tumors showed a modest decrease in CD19+ B-cell infiltration compared with control tumors (Figure S2B). Given that T cells are the primary effectors of bladder cancer immune clearance and their reduced infiltration drives immunotherapy resistance, these data suggest that E6-trunc p53 promotes tumor growth by suppressing intratumoral CD8+/CD4+ T cell recruitment and activation 25-28.

### E6-trunc p53 inhibits innate immune signaling

To elucidate the mechanism by which E6-trunc p53 restrains antitumor immunity, we performed RNA sequencing (RNA-seq) on SW780 cells expressing p53R213*. Differential gene clustering analysis, KEGG enrichment, and Gene Set Enrichment Analysis (GSEA) revealed significant downregulation of pathways associated with IFN-I and cytokines, including Interferon Signaling and Interferon-α/β Signaling (Figure 3A-C). Similar analyses in MB49 cells expressing p53Q189* or p53R210* likewise demonstrated suppression of IFN-associated pathways, including Cytokine Signaling In Immune System (Figure 3D-F). Notably, KEGG analysis also showed enrichment of the RIG-I-like receptor signaling pathway. This finding should not be interpreted as evidence that RNA directly mediates the function of E6-trunc p53. RIG-I-MDA5-mediated RNA sensing and cGAS-STING-mediated DNA sensing share a common downstream TBK1-IRF3-IFN-I signaling module. Thus, suppression of the RIG-I-like receptor pathway in the RNA-seq analysis likely reflects the broader inhibition of PRR-associated IFN-I transcriptional programs caused by E6-trunc p53-mediated TBK1 blockade. qPCR further confirmed these results, showing reduced expression of IFN-β and interferon-stimulated genes (ISGs), such as CCL5, ISG15, CXCL10, and CXCL11 (Figure S2E). ELISA assays revealed diminished secretion of IFN-β, CCL5, and CXCL10 proteins in E6-trunc p53-expressing cells (Figure 3G). These findings indicate that E6-trunc p53 suppresses IFN-I expression in bladder cancer cells.

Previous studies have reported that IFN-I is regulated by innate immune signaling and plays a crucial role in tumor immunity 29. To investigate whether E6-trunc p53 downregulates IFN-I through disruption of innate immune signaling, we performed western blotting of SW780 and MB49 cells expressing either a control vector (PCDH) or E6-trunc p53. Expression of E6-trunc p53 led to decreased phosphorylation of TBK1, IRF3, and STING (Figure 3H). To determine whether the effects of E6-trunc p53 were independent of wild-type p53, we ectopically expressed p53Q192* and p53R213* in p53-null H1299 cells. Notably, both mutants retained the ability to suppress TBK1 phosphorylation (Figure 3H) and IFN-β expression (Figure S2D and 2F) in this p53-deficient background, indicating that E6-trunc p53 exerts a wild-type p53-independent gain-of-function effect. The Western blot analysis and quantitation data for phospho band intensity conducted on the tumor lysates of mice carrying PCDH or p53R210* tumors also showed reduced phosphorylation of TBK1, IRF3 and STING in p53R210* tumors (Figure 3I). The cGAS-STING pathway, which can be activated by dsDNA, induces IFN-I expression 30-32. To assess whether E6-trunc p53 interferes with dsDNA-mediated activation of the STING-TBK1-IRF3 pathway, we transfected control or E6-trunc p53 cells with herring testis DNA (HT-DNA) 33.

In control cells, HT-DNA induced phosphorylation of TBK1, STING, and IRF3, as well as upregulation of IFN-I. However, the response to HT-DNA was markedly attenuated in E6-trunc p53 cells (Figure 3J-K, Figure S3C). After HT-DNA treatment, total STING is often reduced because of lysosomal degradation; however, no such change was observed in our system. To address this discrepancy, we performed a time-course analysis following HT-DNA stimulation (0, 2, 4, 8, 16, and 48 h). Total STING protein levels decreased at early time points and then recovered by 48 h, whereas phosphorylated STING remained detectable (Figure S3A). This suggests that the balance between STING degradation and de novo synthesis may account for the apparent restoration of total STING at the later time point. To further determine whether E6-trunc p53 affects IRF3 activation, we performed immunofluorescence staining to assess IRF3 subcellular localization in SW780 cells. HT-DNA stimulation induced prominent nuclear translocation of IRF3 in control cells, whereas this effect was markedly suppressed in E6-trunc p53-overexpressing cells, supporting the notion that E6-trunc p53 blocks IRF3 nuclear translocation (Figure S3E). Moreover, high-dose HT-DNA induced cell death in control cells, whereas E6-trunc p53 conferred resistance to this effect (Figure S3B-C). These data demonstrate that E6-trunc p53 suppresses innate immune signaling.

### Exon-6-truncated p53 competitively binds to TBK1 and blocks the formation of the TBK1/STING/IRF3 complex

Innate immune signaling is coordinated by multiple sensors, including the dsDNA sensor cGAS-STING, the dsRNA sensors RIG-I/MDA5-MAVS, and toll-like receptors (TLR3 and TLR4), which detect lipopolysaccharides (LPS) 34-36. To investigate how E6-trunc p53 interferes with these pathways, we performed co-immunoprecipitation (co-IP) with an anti-Flag antibody in SW780 cells expressing p53R213* or p53Q192*, followed by Coomassie blue staining of the immunoprecipitated proteins (Figure 4A).

Mass spectrometry analysis identified TBK1 in the immunoprecipitation samples of both p53R213* and p53Q192* (Figure 4A-C), confirming that TBK1 is a central node in innate immune signaling 37, 38. Given prior evidence that mutant p53 binds to TBK1 to suppress innate immunity 19, and considering that both mutant and truncated p53 can alter protein conformation, we hypothesized that E6-trunc p53 may suppress innate immune signaling by sequestering TBK1. Immunofluorescence confirmed the colocalization of E6-trunc p53 with TBK1 in the cytoplasm (Figure 4D). Western blot analysis of the immunoprecipitated (IP) proteins verified that E6-trunc p53 interacted with TBK1, whereas wild-type p53 did not (Figure 4E). Similarly, western blotting of HA-immunoprecipitated (HA-IP) proteins detected E6-trunc p53 (Figure 4F). Using AlphaFold and PyMol, we modeled the structure of E6-trunc and wild-type p53 and predicted its binding sites with TBK1 (Figure 4H). The results showed that the conformation of wild-type p53 protein differs significantly from that of the truncated mutant protein. Although predicted binding sites are present on the wild-type p53 protein, they are fewer in number and distinct from those on the truncated mutant protein. Based on previous studies, we speculate that the inability of wild-type p53 to bind TBK1 in our experimental results may be due to its primary localization in the nucleus, and the formation of wild-type p53 tetramers may obscure potential TBK1 binding sites. These results demonstrate that E6-trunc p53 binds TBK1 in the cytoplasm. TBK1 activation requires formation of a trimeric TBK1-STING-IRF3 complex 38. To determine whether the E6-trunc p53-TBK1 interaction disrupts this assembly, we co-transfected TBK1, STING, and IRF3 into 293T cells expressing either PCDH or E6-trunc p53. In control cells, TBK1 co-immunoprecipitated with both STING and IRF3, whereas E6-trunc p53 expression abolished these interactions (Figure 4G). Overall, these data indicate that E6-trunc p53 interacts with TBK1 to prevent the formation of the trimeric TBK1/STING/IRF3 complex. To determine whether E6-trunc p53 alters cytosolic DNA abundance, we performed immunofluorescence staining for dsDNA in control and E6-trunc p53-expressing cells. No significant difference in cytosolic dsDNA fluorescence intensity was observed between the two groups (Figure 4I), suggesting that E6-trunc p53 does not suppress innate immune signaling by modulating cytosolic DNA content.

### Overexpression of TBK1 restores the type I IFN response suppressed by Exon-6-Truncated p53

To assess whether TBK1 is required for E6-trunc p53-mediated immune suppression, we restored TBK1 expression in E6-trunc p53-expressing cells. Overexpression of TBK1 rescued phosphorylation of STING, TBK1, and IRF3, as well as the type I IFN expression suppressed by E6-trunc p53 (Figure 5A, B; Figure S4A). To determine whether TBK1 overexpression induces apoptosis, we performed TUNEL staining in SW780 and MB49 cells. TBK1 overexpression led to an increase in apoptotic cells, although the magnitude was lower than that observed after HT-DNA treatment, suggesting that TBK1 restoration can partially promote tumor cell apoptosis (Figure S3B). *In vivo*, TBK1 overexpression significantly reduced tumor growth in E6-trunc p53-expressing MB49 cells in syngeneic mice (Figure 5C, D) and restored T cell infiltration in the tumor microenvironment (Figure 5E). Immunofluorescence staining of tissues revealed changes in CD4⁺/CD8⁺ T-cell numbers consistent with the flow cytometry results (Figure 5F). These findings demonstrate that the immunosuppressive effect of E6-trunc p53 can be reversed by restoring TBK1 signaling. These findings further demonstrate that exon-6-truncated p53 blocks activation of innate immune signaling by binding to TBK1.

### Silencing E6-trunc p53 overactivates IFN-I signaling and induces tumor cell death

To assess the role of endogenous TP53 truncating mutations, we examined three cancer cell lines harboring exon 6 truncating mutations of p53: the human fibrosarcoma SW684, the anaplastic carcinoma Calu-6, and the small-cell lung cancer DMS114 (Figure S4B). RNA-seq following knockdown of E6-trunc p53 in DMS114 cells revealed an enrichment of differentially expressed genes in type I IFN and adaptive immune pathways, including Interferon Alpha/Beta Signaling and the Innate Immune System (Figure 6A-C). Consistently, Western blotting showed that knockdown of E6-trunc p53 enhanced the phosphorylation of STING, TBK1, and IRF3 in both Calu-6 and DMS114 cells (Figure 6D). qPCR and ELISA further confirmed the increased expression of IFN-β and multiple interferon-stimulated genes (ISGs) (Figure 6E, F; Figure S4C, D). Previous studies have reported that activation of the innate immune response leads to IRF3 phosphorylation and subsequent nuclear translocation 39. Immunofluorescence demonstrated nuclear accumulation of IRF3 following E6-trunc p53 knockdown (Figure S4E). These results indicate that knockdown of E6-trunc p53 activates innate immune signaling.

E6-trunc p53 knockdown also impaired cell viability. Crystal violet staining showed reduced cell viability after E6-trunc p53 knockdown (Figure S4F). Consistently, PI-based live/dead staining revealed an increased fraction of dead cells, although the Calcein signal varied across cell lines and was used mainly as supportive evidence (Figure S4G). More importantly, Annexin V/7AAD flow cytometry independently confirmed that silencing E6-trunc p53 induced marked apoptosis in Calu-6 and DMS114 cells (Figure 6G).

To determine whether innate immune activation and cell death in E6-trunc p53 knockdown cells depend on TBK1, we performed a combined knockdown of TBK1 and E6-trunc p53. Depletion of E6-trunc p53 enhanced the phosphorylation of STING, TBK1, and IRF3, while co-silencing TBK1 abrogated this activation (Figure 6H). qPCR and ELISA confirmed that IFN-β expression returned to baseline levels in the double-knockdown cells (Figure 6I, Figure S5B). To determine whether the IFN-β response regulated by E6-trunc p53 depends on the cGAS-STING pathway, we performed combined knockdown experiments in Calu-6 cells. E6-trunc p53 depletion alone significantly increased IFN-β expression and activated TBK1, IRF3, and STING phosphorylation. By contrast, co-silencing of cGAS or STING markedly blunted these effects, indicating that E6-trunc p53 regulates type I interferon signaling in a cGAS-STING-dependent manner (Figure 6J, Figure S5C-D). Flow cytometry demonstrated that TBK1 knockdown significantly rescued cell death induced by E6-trunc p53 knockdown (Figure 6K; Figure S5A). To determine whether cell death was linked to IFN-I activation resulting from TBK1 activation, we performed IFNB-knockout. Deletion of IFNB restored cell viability, as shown by crystal violet assays, live/dead staining, and flow cytometry (Figure 6L; Figure S6A-D). These results suggest that cell death induced by E6-trunc p53 silencing requires both TBK1 and IFN-β. Collectively, these data demonstrate that silencing E6-trunc p53 activates innate immune signaling through TBK1 and induces cell death, further confirming that E6-trunc p53 suppresses innate immunity by inhibiting TBK1.

### Truncated p53 confers resistance to PD-1 immunotherapy in bladder cancer

Immune checkpoint inhibitors (ICIs) targeting PD-1/PD-L1 are increasingly being applied in advanced bladder cancer 40-42. Investigating the mechanisms of resistance to ICIs and improving their efficacy is of significant clinical importance. Given that E6-trunc p53 suppresses CD8+ and CD4+ T cell infiltration and activation, we investigated whether it compromises the preclinical efficacy of ICIs. In syngeneic MB49 models, anti-PD-1 treatment markedly inhibited tumor growth in control tumors and reduced tumor weight by approximately 76% in the control group, whereas its inhibitory effect on p53R210* tumors was weaker, leading to only about a 50% reduction in tumor weight (Figure 7A; Figure S7A). Flow cytometry and immunofluorescence confirmed that PD-1 blockade increased CD8+ and CD4+ T cell infiltration, yet E6-trunc p53 suppressed T cell numbers regardless of treatment (Figure 7B-C). Body weight remained unchanged across groups, indicating treatment tolerability (Figure S7B). To better recapitulate the native bladder tumor microenvironment, we performed anti-PD-1 therapy experiments in orthotopic bladder tumor models. Similarly, E6-trunc p53 tumors resisted PD-1-mediated growth suppression (Figure 7D, E). These findings demonstrate that E6-trunc p53 abrogates the antitumor efficacy of ICIs.

To evaluate the clinical relevance, we analyzed surgical specimens from 349 bladder cancer patients across two centers: 301 Hospital and Third Central Hospital. Based on previous classifications of p53 genotype-specific immunohistochemical (IHC) characteristics 20, 21, we screened bladder cancer cases for potential E6-trunc p53 using immunohistochemistry. Then we included cases showing a detectable truncated p53 band within a relatively similar molecular-weight range, close to the expected size of exon-6-truncated p53. Samples with ambiguous, highly heterogeneous, or multiple inconsistent p53 bands were excluded from the truncated p53-positive cohort. Using this strategy, 32 patients were classified as truncated p53-positive (Figure 7F; Figure S7C). Kaplan-Meier analysis revealed significantly poorer overall survival in truncated p53 patients compared with wild-type cases (Figure 7G).

To further evaluate the impact of truncated p53 on ICI treatment, multiplex immunofluorescence further demonstrated reduced CD8+ and CD4+ T cell infiltration in truncated p53 tumors compared with wild-type tumors (Figure 7H). We identified 16 patients with truncated p53 and 81 patients with wild-type p53 who received ICIs. Truncated p53 patients exhibited a significantly higher rate of non-response compared to wild-type cases (Figure 7I, J), which also showed shorter progression-free survival (Figure 7K). In conclusion, these data establish E6-trunc p53 as a potential biomarker of ICIs resistance and poor prognosis in bladder cancer, highlighting the inhibition of E6-trunc p53 as a promising therapeutic strategy.

## Discussion

As the best-characterized tumor suppressor, p53 plays a pivotal role in cancer development, progression, prognosis, and resistance to both immunotherapy and chemotherapy 43. TP53 truncating mutations, second in frequency only to point mutations, are generally considered loss-of-function alterations 10, 12. In contrast to prevailing views, a prior study revealed that E6-trunc p53 exerts pro-metastatic effects through interaction with cyclophilin D within mitochondria 44. In the present study, we found that E6-trunc p53 suppresses the cGAS-STING-IFN-I innate immune signaling pathway to drive both cell-intrinsic and cell-extrinsic oncogenic activities. Moreover, multiple studies indicate that TP53 truncating mutations are associated with worse disease-free and overall survival across various cancer types 20, 21. Our data further showed that E6-trunc p53 is an independent prognostic marker for poor outcomes in MIBC. Furthermore, patients harboring TP53 truncating mutations exhibit markedly reduced T-cell infiltration and diminished responses to immunotherapy. We therefore propose that E6-trunc p53 may have acquired a separation-of-function property that fuels malignancy. In cells carrying endogenous E6-trunc p53, combined knockdown of cGAS/STING markedly attenuated shP53-induced upregulation of IFN-I, indicating that E6-trunc p53 does not act independently of upstream innate immune sensing. Rather, it appears to suppress type I interferon signaling in a cGAS-STING-dependent manner, thereby limiting TBK1 activation and downstream IRF3 signaling.

Recent studies have shown that mutant p53 can regulate macrophage polarization within the tumor microenvironment 19. Consistent with this concept, our findings suggest that E6-trunc p53 may also influence macrophage behavior by promoting M2-like polarization, as evidenced by the increased number of CD206-positive macrophages in xenograft tumors. This effect may contribute to the establishment of an immunosuppressive microenvironment, potentially as a consequence of suppressed type I interferon signaling. Further studies are warranted to elucidate the underlying molecular mechanisms and to determine whether E6-trunc p53 directly regulates macrophage recruitment or polarization.

Because no bladder cancer cell lines were available with TP53 exon-6 truncating mutations, we obtained other human tumor cell lines harboring these mutations as reported previously 44. Silencing of endogenous E6-trunc p53 induced strong activation of the cGAS-STING-IRF3 axis, which in turn led to a pronounced upregulation of IFN-I signaling. Concurrently, we observed increased cell death. Mounting evidence indicates that excessive activation of the IFN-I pathway can induce cell death 45, 46. As expected, deletion of TBK1 or IFN-β substantially attenuated the cell death induced by knockdown of the E6-trunc p53. However, further research is required to elucidate the cell death-inducing mechanism underlying E6-trunc p53 knockdown, which may help clarify its unique functional role. The inclusion of cGAS in our Western blot analyses helped clarify whether the E6-deficient p53 only functions at the level of TBK1. However, the more substantial alterations in p-TBK1 and p-STING suggest that the primary inhibitory effect occurs downstream of cGAS activation. We speculate that the observed changes in cGAS expression may reflect secondary regulation by type I interferon signaling, which is known to reinforce cGAS transcription through a positive feedback loop. Overall, even though E6-trunc p53 lacks transcriptional activity and canonical tumor-suppressive functions, it remains capable of reprogramming cellular signaling networks and modifying cellular dependencies and states.

Recent studies have shown that mutant p53 can promote cytosolic DNA accumulation and activate non-canonical NF-κB signaling 47, whereas wild-type p53 has also been reported to regulate cytosolic DNA and stimulate IRF3-mediated type I interferon responses 48. In contrast, our data indicate that exon-6-truncated p53 does not significantly alter cytosolic dsDNA abundance. Instead, E6-trunc p53 suppresses innate immune signaling through direct interaction with TBK1, thereby inhibiting IFN-I production. Together, these findings suggest that different p53 isoforms and mutation classes may engage distinct mechanisms to modulate cytosolic nucleic acid sensing and downstream inflammatory signaling.

The traditional view holds that truncated p53 isoforms are functionally deficient, producing unstable proteins prone to degradation and therefore difficult to detect 10, 12. However, analyses of cell lines and clinical samples revealed that E6-trunc p53 isoforms are primarily localized in the cytoplasm and can be persistently expressed, thereby establishing the basis for their functional activity. Previous studies have demonstrated that mutant p53, but not wild-type p53, can bind to TBK1 19. However, results from several studies showed that the majority of missense mutant p53 remains localized in the nucleus, with only a small fraction found in the cytoplasm 19. Our findings suggest that E6-trunc p53 is also capable of binding tightly to TBK1, which may be related to the inability of the protein to enter the nucleus and form tetramers due to the C-terminal deletion. Further mapping or structural analyses could help elucidate the underlying mechanism. These effects persist in p53-null cells and are also observed in endogenous E6-trunc p53-expressing cell lines lacking wild-type p53. Together, these findings suggest that E6-trunc p53 may function as a wild-type p53-independent oncogenic factor rather than solely acting through dominant-negative inhibition. Furthermore, our immunocompetent mouse model combined with patient cohort analysis clarified the weakening effect of E6-trunc p53 on PD-1 blockade efficacy and its molecular basis. Therefore, developing small-molecule inhibitors or degraders targeting E6-trunc p53 based on structural analysis may provide new strategies to overcome resistance to immunotherapy.

The sample size of patients with TP53 exon 6 truncation mutations in this study is relatively limited, which may diminish the statistical power of the analysis and affect the representativeness of the results for a broader bladder cancer population. Future studies should involve larger-scale, multi-center, prospective patient cohorts to improve statistical power and clinical relevance. Due to the absence of murine tumor cells expressing E6-trunc p53, our *in vivo* tumor immunity research has been limited to overexpression models. Future studies generating transgenic mouse models expressing E6-trunc p53 will be essential to evaluate its contribution to tumorigenesis and tumor immune escape, and to verify whether targeting E6-trunc p53 can enhance the recognition and cytotoxicity of immune effector cells against tumors.

A limitation of this study is that endogenous TP53 exon-6 truncating mutations were not generated by CRISPR/Cas9-mediated knock-in in bladder cancer cells. Although such an approach would provide an ideal model to assess the function of E6-trunc p53 in its native genomic context, the lack of available bladder cancer cell lines harboring endogenous exon-6 TP53 truncations, together with the technical challenges associated with introducing precise truncating mutations and obtaining multiple independently validated clones, precluded this strategy within the present study. To partially overcome this limitation, we examined additional cancer cell lines that naturally carry TP53 exon-6 truncating mutations and observed results consistent with those from our ectopic expression models. Future studies using CRISPR knock-in and genetically engineered mouse models will be important to further validate these findings.

In conclusion, our study demonstrates that TP53 exon 6 truncation mutations suppress IFN-I-mediated antitumor immune responses by binding to TBK1, thereby driving bladder cancer progression and conferring resistance to PD-1-based immunotherapy. These findings implicate TP53 exon 6 truncation mutations as potential biomarkers and therapeutic targets, laying a theoretical foundation for refining bladder cancer immunotherapy strategies.

## Methods

### Sex as a biological variable

Human bladder cancer samples from both male and female patients were analyzed. Both male and female mice were included in all experimental studies. In this study, sex was not analyzed as a biological variable.

### Mouse strains

Six- to eight-week-old C57BL/6J mice and BALB/c nude mice were purchased from Belli Biology Co., Ltd (Beijing, China). For all experiments, age-matched cohorts of male or female mice (6-8 weeks old) were employed. Experimental and control animals were either littermates or were co-housed for at least one week before experimentation, after which they were randomly assigned to experimental groups. All procedures were performed with mice housed under specific pathogen-free (SPF) conditions. Animals had ad libitum access to food and water and were maintained on a standard 12-hour light/dark cycle at room temperature.

### Cell lines

The SW780 bladder cancer cell line, HEK293T cell line, SW684 human fibrosarcoma cell line, and Calu-6 human carcinoma cell line were obtained from ATCC. The DMS 114 human small cell lung cancer cell line was sourced from Cellverse Co., Ltd. (Shanghai, China). The MB49 mouse malignant bladder cell line was procured from Zhejiang Maizen CTCC, China. UMUC3, SW780, Calu-6, and MB49 cells were cultured in DMEM (Procell, China), while SW684 cells were maintained in MEM (Procell, China). DMS 114 cells were cultured in RPMI-1640 (Procell, China). All culture media were supplemented with 10% fetal bovine serum (Procell, China) and 1% penicillin-streptomycin (Procell, China). Cells were incubated under conditions of 37 °C and 5% CO2. All cell lines were regularly tested and confirmed to be free of mycoplasma contamination.

### Clinical samples

Postoperative human bladder cancer tissues and the corresponding clinical data were obtained from the Departments of Urology at 301 Hospital and the Third Central Hospital. The histology and pathological subtypes of all specimens were independently confirmed by three experienced pathologists. Among 632 bladder cancer patients across the two centers, 175 received treatment with immune checkpoint inhibitors (ICIs), and 349 patients were followed. The therapeutic efficacy of ICIs was assessed by at least three pathologists according to the Response Evaluation Criteria in Solid Tumors (RECIST). Written informed consent was obtained from all patients prior to study initiation.

### Plasmid construction and lentivirus production

Biomed (Beijing) synthesized short hairpin RNA (shRNA) sequences targeting human TP53 and TBK1, which were subsequently cloned into the pLKO vector for subsequent experiments. Full-length cDNAs of human TP53, P53Q192*, P53R213*, TBK1, STING, and IRF3, along with murine Trp53, p53Q189*, p53R210*, and Tbk1, were synthesized by Biomed and subsequently cloned into the PCDH vector to construct overexpression plasmids. The IFN-β sgRNA plasmid was procured from Miaoling Co., Ltd. (Beijing, China). Lentiviral vectors were transfected into HEK293T cells using PAX2 and VSVG packaging plasmids. After 48 hours, supernatants were collected and concentrated overnight at 4 °C using the Lentivirus Concentration Reagent (GenStar, China). To establish stable transduced cell lines, cells were infected with lentivirus for 48 hours, followed by selection with puromycin or appropriate drugs for one week. The shRNA sequences are provided in Table S1.

### Cell viability assay

For cell proliferation assays, bladder cancer cells were seeded into 96-well plates (2000 cells per well). Cell viability was measured at various time points using the Cell Counting Kit-8 (Dojindo, Japan), in accordance with the manufacturer's instructions.

### Colony formation assay

Cells were seeded into six-well plates (300 cells per well). Complete medium was replaced every other day, and colony growth was continuously monitored. When an individual colony contained more than 50 cells, the cells were washed and fixed, stained with crystal violet for 20 minutes, gently rinsed with running water to remove excess dye, and the results were recorded for statistical analysis.

### qRT-PCR

RNA was extracted using the Direct-zol RNA MiniPrep Kit (Zymo Research, R2062), following the manufacturer's protocol. Reverse transcription to cDNA was conducted using the High-Capacity cDNA Reverse Transcription Kit (Life Technologies, 4374966) with 1 μg of RNA. Quantitative PCR was conducted using a Bio-Rad CFX 9600 system and SYBR Green PCR Master Mix (Life Technologies, A25778). Relative cDNA quantification was performed using target gene primers and internal control primers, as listed in Table S1.

### Western blot analysis

Cells subjected to different treatments were lysed on ice for 30 min using RIPA lysis buffer supplemented with PMSF, followed by centrifugation at 12,000 × g for 10 min at 4 °C. The supernatants were collected, and total protein concentration was determined using a BCA protein assay kit. An equal amount of protein from each sample was mixed with 5× SDS loading buffer and denatured at 80-95 °C for 10 min. Proteins were separated by 10% SDS-PAGE and subsequently transferred onto PVDF membranes. The membranes were blocked with 5% non-fat milk in TBST at room temperature for 1 h, and then incubated with the appropriate primary antibodies at 4 °C overnight with gentle agitation. After washing three times with TBST (5 min each), the membranes were incubated with the corresponding secondary antibodies at room temperature for 1 h. The membranes were then washed three additional times with TBST and visualized using an enhanced chemiluminescence (ECL) detection system. Protein bands were captured using a chemiluminescence imaging system. All experiments were independently repeated three times.

### Flow cytometry analysis of tumor-infiltrating immune cells

Mouse tumors were collected, weighed, minced, and enzymatically digested at 37 °C for 60 minutes with DNase I (Solarbio, China) and collagenase IV (Sigma, USA). After digestion was terminated with RPMI-1640 medium, the dissociated cells were filtered through a 70 μm cell strainer (BIOFIL, China) to obtain a single-cell suspension for flow cytometry analysis. Red blood cells were lysed using RBC Lysis Solution (Beyotime, China). The cells were stained with BV510 anti-mouse CD45 (Biolegend, 103137, USA), BV605 anti-mouse CD3 (Biolegend, 100237, USA), Alexa Fluor®488 anti-mouse CD4 (Biolegend, 100423, USA), APC/Fire™750 anti-mouse CD8a (Biolegend, 100766, USA), and APC anti-mouse NK1.1 (Biolegend, 108710, USA) for 30 minutes at room temperature, protected from light. The stained cells were analyzed by flow cytometry using FlowJo software 18.

### *In vitro* flow cytometry

For *in vitro* flow cytometry, cells were digested, resuspended, washed with PBS, and stained with an apoptosis detection kit for 30 min at room temperature.

### ELISA

Cell culture supernatants were collected from 6-well plates, and levels of human IFN-β, CCL5, and CXCL10 were quantified using ELISA kits (Elabscience, USA), following the manufacturer's instructions. The concentration of cytokines/chemokines was determined based on OD values at a wavelength of 450 nm.

### Co-immunoprecipitation (Co-IP)

HEK293T cells were transfected with FLAG-tagged P53R213* or P53WT and HA-TBK1 for 48 hours, followed by co-immunoprecipitation. Cells were lysed in NP-40 lysis buffer (50 mM Tris-HCl, pH 7.4; 150 mM NaCl; 1% NP-40) supplemented with protease and phosphatase inhibitors. Lysates were centrifuged at 12,000 × g for 15 min at 4 °C and pre-cleared with protein A/G agarose beads. Pre-cleared supernatants were incubated overnight at 4 °C with primary antibodies or species-matched IgG controls (2 μg per 500 μg total protein) under constant rotation. Immune complexes were captured with protein A/G agarose beads for 2 h at 4 °C. Beads were washed four times with lysis buffer, and bound proteins were eluted in 2× Laemmli buffer by boiling at 95 °C for 5 min. Eluted proteins were analyzed by western blotting using FLAG (Proteintech, 66008-4-Ig, USA) and HA (Proteintech, 51064-2-AP, USA) antibodies.

### Mass spectrometry analysis

#### Protein digestion and desalination

Cut the gel strip into small pieces approximately 1 mm³ in size using a scalpel and place them into a 1.5 mL centrifuge tube. Add decolorization solution and incubate at 60 °C with shaking. Repeat this process multiple times until the gel becomes transparent. Add acetonitrile to dehydrate the gel pieces until they turn white, then dry under vacuum. Add an appropriate amount of 10 mM TCEP and 25 mM CAA (final concentrations) and react at 95 °C for 10 minutes. Again, add acetonitrile to dehydrate the gel pieces until they turn white, dry under vacuum, and then add deionized water for washing. Repeat this step once. Add 50 mM ammonium bicarbonate and incubate for 10 minutes. Then add trypsin working solution, ensuring full contact between the enzyme solution and the gel pieces. After the enzyme solution is completely absorbed by the gel pieces, incubate at 37 °C overnight. The next day, centrifuge to collect the enzymatic supernatant and transfer it to a new centrifuge tube. Add acetonitrile to the remaining gel pieces, vortex for 5 minutes, centrifuge to collect the enzymatic supernatant, and combine it with the previous supernatant. Add 0.1% formic acid (FA) to the remaining gel pieces to terminate the reaction. After reabsorption of liquid, add acetonitrile and vortex for 5 minutes. Collect the enzymatic supernatant and combine it with the previous supernatant. Freeze-dry the combined supernatant. Desalt the sample using a C18 desalting column. Activate the desalting column with 100% acetonitrile, equilibrate it with 0.1% formic acid, and load the sample onto the column. Then wash the column with 0.1% formic acid to remove impurities. Finally, elute with 40% acetonitrile, collect the flow-through liquid, and freeze-dry it.

#### LC-MS/MS analysis

Nanoflow LC-MS/MS analysis of tryptic peptides was conducted on a quadrupole Orbitrap mass spectrometer (Q Exactive HF-X, Thermo Fisher Scientific, USA) coupled to an EASY nLC 1200 ultra-high pressure system (Thermo Fisher Scientific) via a nano-electrospray ion source.1 μg of peptides were loaded on a 25 cm column (100 μm inner diameter, packed using ReproSil-Pur C18-AQ 1.5- µm silica beads; QL-HPLC-100*1.5; Beijing Qinglian Biotech Co., Ltd, Beijing, China). Peptides were separated using a gradient from 8 to 12% B in 2 min, then 12% to 30 % B in 3 min and stepped up to 40% in 7 min followed by a 10 min wash at 95% B at 300 nl/minute where solvent A was 0.1% formic acid in water and solvent B was 80% ACN and 0.1% formic acid in water. The total duration of the run was 30 min. Column temperature was kept at 60 °C using an in-house-developed oven. Briefly, the mass spectrometer was operated in “top-40” data-dependent mode, collecting MS spectra in the Orbitrap mass analyzer (120,000 resolution, 350-1500 m/z range) with an automatic gain control (AGC) target of 3E6 and a maximum ion injection time of 80 ms. The most intense ions from the full scan were isolated with an isolation width of 1.6 m/z. Following higher-energy collisional dissociation (HCD) with a normalized collision energy (NCE) of 27, MS/MS spectra were collected in the Orbitrap (15,000 resolution) with an AGC target of 5E4 and a maximum ion injection time of 45 ms. Precursor dynamic exclusion was enabled with a duration of 16 s.

#### Identification and quantitation of protein

All RAW files were analyzed using the Proteome Discoverer suite (version 2.4, Thermo Fisher Scientific). MS2 spectra were searched against the UniProtKB human proteome database containing both Swiss-Prot and TrEMBL human reference protein sequences (20,434 target sequences downloaded on 7 March 2024). The Sequest HT search engine was used, and parameters were specified as follows: fully tryptic specificity, maximum of two missed cleavages, minimum peptide length of 6, fixed carbamidomethylation of cysteine residues (+57.02146Da), variable modifications for oxidation of methionine residues (+15.99492Da), precursor mass tolerance of 15 ppm and a fragment mass tolerance of 0.02Da for MS2 spectra collected in the Orbitrap. Percolator was used to filter peptide spectral matches and peptides to a false discovery rate (FDR) of less than 1%. After spectral assignment, peptides were assembled into proteins and were further filtered based on the combined probabilities of their constituent peptides to a final FDR of 1%. As default, the top matching protein or 'master protein' is the protein with the largest number of unique peptides and with the smallest value in the percent peptide coverage (that is, the longest protein). Only unique and razor (that is, parsimonious) peptides were considered for quantification. The mass spectrometry analysis results are presented in Table S2.

### Immunohistochemical staining (IHC)

Paraffin-embedded tissue sections (5 μm) fixed in 4% PFA were deparaffinized and rehydrated. Heat-induced antigen retrieval was carried out in citrate buffer (pH 6.0). Endogenous peroxidase activity was quenched with 3% H₂O₂, and non-specific binding sites were blocked with 10% normal goat serum. Sections were incubated overnight at 4 °C with primary antibodies diluted in blocking serum according to the manufacturer's instructions, followed by incubation with species-matched enzyme-conjugated secondary antibodies for 1 h at room temperature. Signals were developed with a DAB substrate, and sections were counterstained with hematoxylin. Slides were dehydrated, cleared in xylene, and mounted with synthetic resin. Imaging was performed with a bright-field microscope (Nikon Eclipse Ci-L).

### Immunofluorescence

For cell immunofluorescence, cells were seeded on 14-mm glass coverslips, washed three times with PBS, fixed with 4% paraformaldehyde for 20 minutes at room temperature, and permeabilized with 0.3% Triton-X100 in PBS for 3 minutes at room temperature. Cells were blocked with 1% BSA and incubated overnight at 4 °C with primary antibody A. The following day, cells were washed three times with PBST and incubated with secondary antibody A (ABflo®594-conjugated goat anti-mouse IgG, Abclone, AS054, China) for 1 hour at room temperature. For protein colocalization experiments, additional primary and secondary antibodies were applied, followed by mounting with an anti-fade mounting medium containing DAPI.

For multiplex immunofluorescence staining of tissues, 5-μm sections from formalin-fixed, paraffin-embedded (FFPE) tissue blocks were processed according to a standardized multiplex immunofluorescence protocol. Following deparaffinization and antigen retrieval (citrate buffer, pH 6.0, 95 °C, 20 min), sections were sequentially incubated with primary antibodies. Each staining cycle consisted of primary antibody incubation (2 h, RT), incubation with HRP-conjugated secondary antibody (30 min, RT), and tyramide signal amplification (TSA) using fluorophore-conjugated tyramide (10 min, RT). Between staining cycles, antibodies were stripped with glycine-HCl buffer (pH 2.0). Tissue autofluorescence was quenched with Sudan Black B (0.1% in 70% ethanol). Cell nuclei were counterstained with DAPI (5 min, RT), and slides were mounted in anti-fade medium. Multispectral imaging was conducted on a confocal microscope (Zeiss LSM 900) using uniform exposure settings across all samples. For each tissue sample, 6 microscopic fields at 200× magnification were selected; the number of T cells in each field was recorded and subjected to statistical analysis.

### Animal studies

Subcutaneous tumor formation was conducted in 6-8-week-old C57BL/6J and BALB/c nude mice. MB49 cells (1×10^6 cells per mouse) stably transfected with plasmid were injected subcutaneously into the right flank of each mouse. Tumor volume was measured every two days using calipers and calculated as Volume = Length × Width² × 0.5. For immune therapy resistance experiments, 6-week-old C57BL/6J mice were subcutaneously inoculated with MB49 cells (1×10^6 cells per mouse). On day 5, mice were intraperitoneally injected with 100 μg anti-PD-1 antibody (BioXcell, BE0241, USA) every three days 49.

#### Orthotopic bladder tumor model

A mouse model of orthotopic bladder cancer was developed. Briefly, under anesthesia, C57BL/6J mice were positioned in a supine posture. A 1 cm midline incision was made on the skin over the abdominal pelvic region, and the bladder was exposed by dissecting the abdominal muscles. Luciferase-labeled MB49 cells, resuspended in 50% Matrigel (LabelLead, China) and PBS (5×10^5 cells per mouse), were injected into the bladder wall using an insulin syringe. The bladder was returned to its original position, and the abdominal muscles and skin were sutured. Mice were allowed to recover on a heating pad. For bioluminescent imaging, mice were intraperitoneally injected with 200 μL (15 mg/ml) D-luciferin potassium salt (LabelLead, China) and imaged using a real-time imaging system (Berthold LB983, Germany) 50. All animal experiments were approved by the Institutional Animal Use and Care Committee and granted approval with AP# GPT-BJAP001.

#### RNA-seq and Gene Set Enrichment Analysis (GSEA)

Total RNA was extracted using the MJZol Total RNA Extraction Kit (Majorbio, China). RNA-seq analysis was conducted by Majorbio Biopharmaceutical Technology Co., Ltd. (Shanghai, China). The transcriptomic library was prepared using the TruSeq RNA Sample Preparation Kit (Illumina, San Diego, CA) with 1 μg of total RNA. Differentially expressed genes (DEGs) with |log2FC| > 1.5 and P-adjusted values ≤ 0.05 were considered statistically significant. Data were analyzed using the Majorbio cloud platform, and sequencing results were deposited in the Gene Expression Omnibus (GEO) database (GSE308506), accession number spmfoymcptazzsd. GSEA was conducted using the Molecular Signatures Database (MSigDB) to analyze the biological functions of genes associated with IFN-I signaling.

### Statistical analysis

Data are presented as mean ± SD. Overall survival was calculated using the Kaplan-Meier method and the log-rank test. Differences between groups were analyzed using two-tailed t-tests, one-way ANOVA with Dunnett's multiple comparisons test, or two-way ANOVA. Statistical significance was assessed using GraphPad Prism software, with P < 0.05 considered statistically significant. ns, not significant, *P < 0.05, **P < 0.01, ***P < 0.001.

### Study approval

All bladder cancer tissue samples used in this study were obtained with informed consent from patients and approved by the Institutional Review Board of the General Hospital of the People's Liberation Army. All animal experiments were conducted in accordance with protocols approved by the Animal Care and Use Committee of the General Hospital of the People's Liberation Army.

## Supplementary Material

Supplementary figures and table 1.

Supplementary table 2.

## Figures and Tables

**Figure 1:**
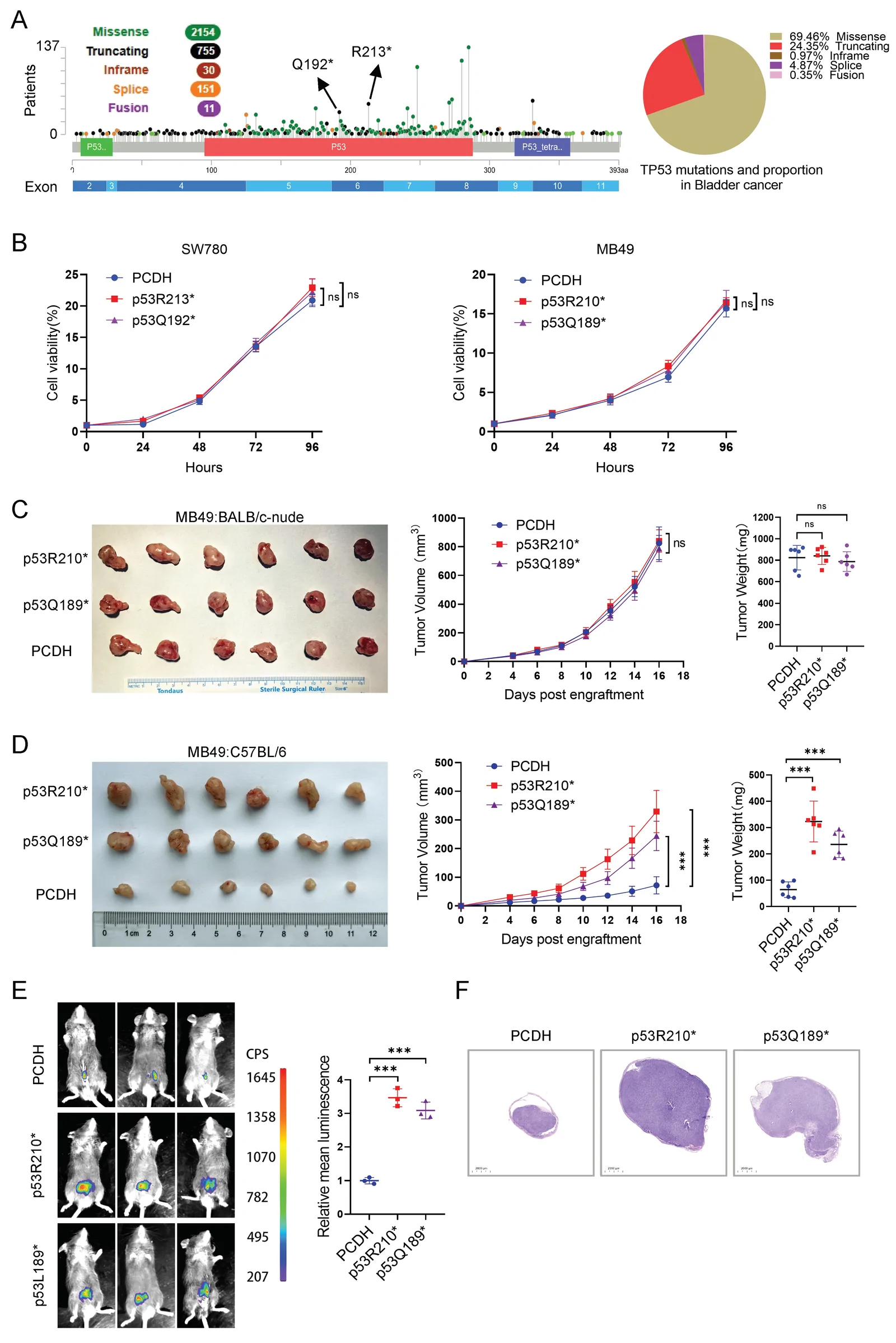
** Overexpression of E6-trunc p53 promotes tumor growth in immunocompetent mice.** (A) TP53 gene mutation landscape in bladder cancer samples from the TCGA, MSK, MDACC, BGI, and IGMBC databases. (B) Cell viability of SW780 and MB49 cells stably transfected with PCDH or E6-trunc p53, measured using the CCK-8 assay at 0, 24, 48, 72, and 96 hours. (C) Tumor images, growth curves, and tumor weights of immunodeficient nude mice (n = 6) subcutaneously injected with PCDH or E6-trunc p53 MB49 cells. (D) Tumor images, growth curves, and tumor weights of immunocompetent C57BL/6J mice (n = 6) subcutaneously injected with PCDH or E6-trunc p53 MB49 cells. (E) Representative bioluminescent images and histogram analysis of bioluminescence intensity in C57BL/6J mice (n = 6) with bladder orthotopic injection of luciferase-labeled PCDH or E6-trunc p53 MB49 cells. (F) Representative hematoxylin and eosin (H&E) staining of bladder orthotopic tumors. Scale bars, 2 mm. Data were presented as mean ± SD. Statistical significance for panels C, D, and E was determined using a two-tailed unpaired Student's t-test, while panel B utilized two-way ANOVA. ns, not significant. **p < 0.01, ***p < 0.001.

**Figure 2:**
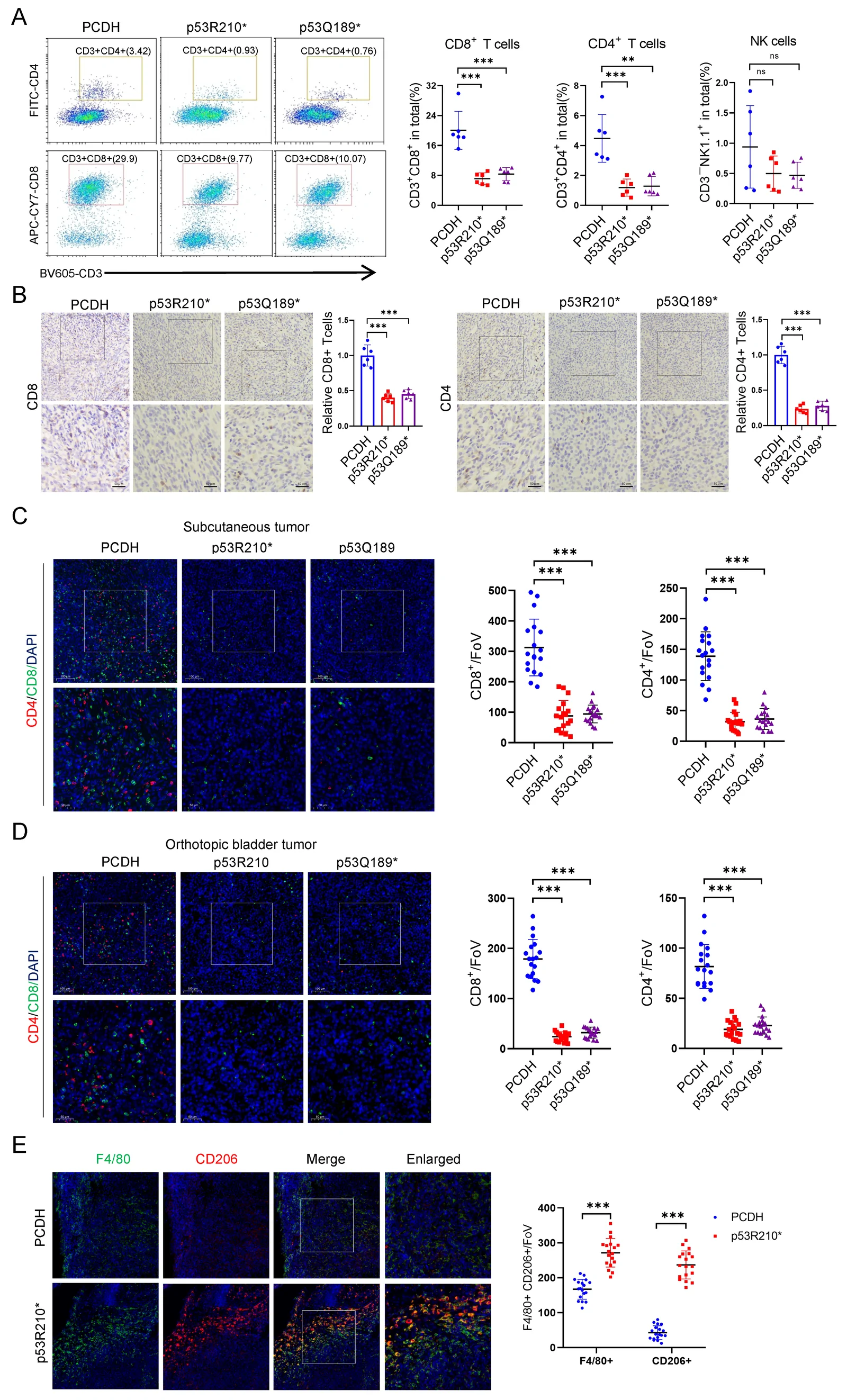
** Overexpression of E6-trunc p53 attenuates intratumoral CD8⁺ and CD4⁺T cells.** (A) Flow cytometric analysis of tumor-infiltrating CD8+ T cells, CD4+ T cells, and NK cells in E6-trunc p53-MB49 tumors transplanted into C57BL/6J mice (n = 6). (B) Representative immunohistochemical staining and positive cell counts for CD8 and CD4 in PCDH or E6-trunc p53 MB49 subcutaneous tumors (n = 6). Scale bars, 50 μm. (C) Representative images and immunofluorescence quantification of CD8 and CD4 in PCDH or E6-trunc p53 MB49 subcutaneous tumors (n = 6). Scale bars, 50 μm. (D) Representative images and immunofluorescence quantification of CD8 and CD4 in PCDH or E6-trunc p53 MB49 bladder orthotopic tumors (n = 6). Scale bars, 50 μm. Data were presented as mean ± SD. Statistical significance for panels A, B, C, and D was determined using a two-tailed unpaired Student's t-test. ns, not significant. **p < 0.01, ***p < 0.001. (E) Double immunofluorescence staining for F4/80 and CD206 showed increased M2-like macrophage polarization in E6-trunc p53-overexpressing xenografts.

**Figure 3:**
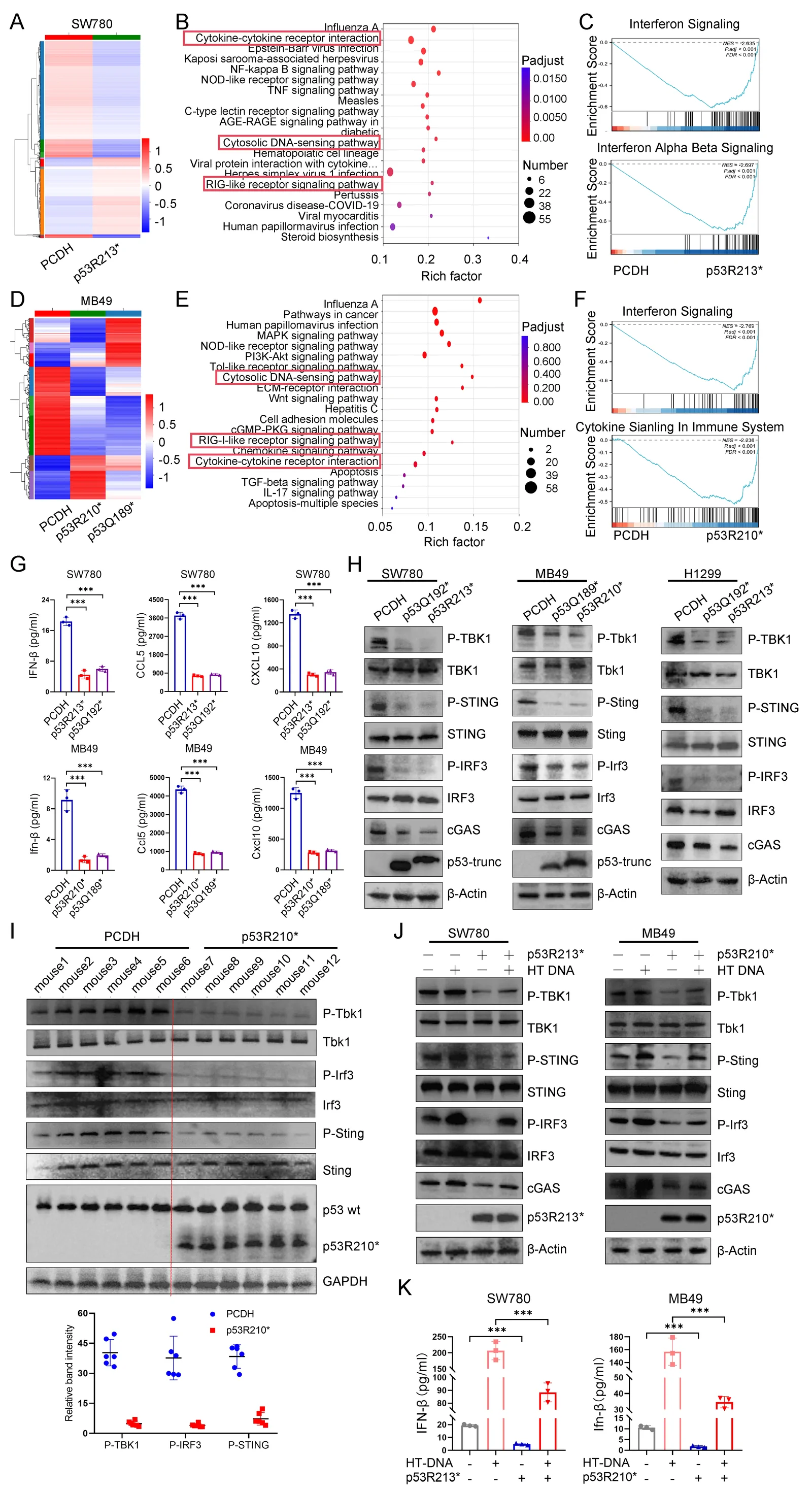
** E6-trunc p53 suppresses innate immune signaling.** (A) Unsupervised clustering heatmap depicted differentially expressed mRNAs in PCDH and p53R213* SW780 cells. (B) The top 20 significantly enriched KEGG pathways in the indicated SW780 cells. (C) Representative GSEA plots of innate immunity-related gene sets in the indicated SW780 cells. (D) Unsupervised clustering heatmap depicted differentially expressed mRNAs in PCDH, p53Q189* and p53R210* MB49 cells. (E) The top 20 significantly enriched KEGG pathways in the p53Q189* and p53R210* MB49 cells. (F) Representative GSEA plots of innate immunity-related gene sets in the indicated MB49 cells. (G) ELISA assay showed the expression levels of IFN-β, CCL5, and CXCL10 proteins in the indicated SW780 and MB49 cells. (H) Western blot analysis of PCDH or E6-trunc p53 in SW780 MB49 and H1299 cells. (I) Western blot analysis of PCDH or E6-trunc p53 in MB49 tumor tissues (n = 6). Densitometric quantification of phosphorylated bands (pTBK1, pSTING, and pIRF3) was normalized to GAPDH and is shown below the representative Western blots. (J) Immunoblot analysis of SW780 and MB49 cells treated with 4 μg/ml HT-DNA for 48 hours. (K) ELISA assay showed the expression levels of IFN-β protein in the indicated SW780 and MB49 cells. Data were presented as mean ± SD. Statistical significance for panels G, I, and K was determined using a two-tailed unpaired Student's t-test. ***p < 0.001.

**Figure 4:**
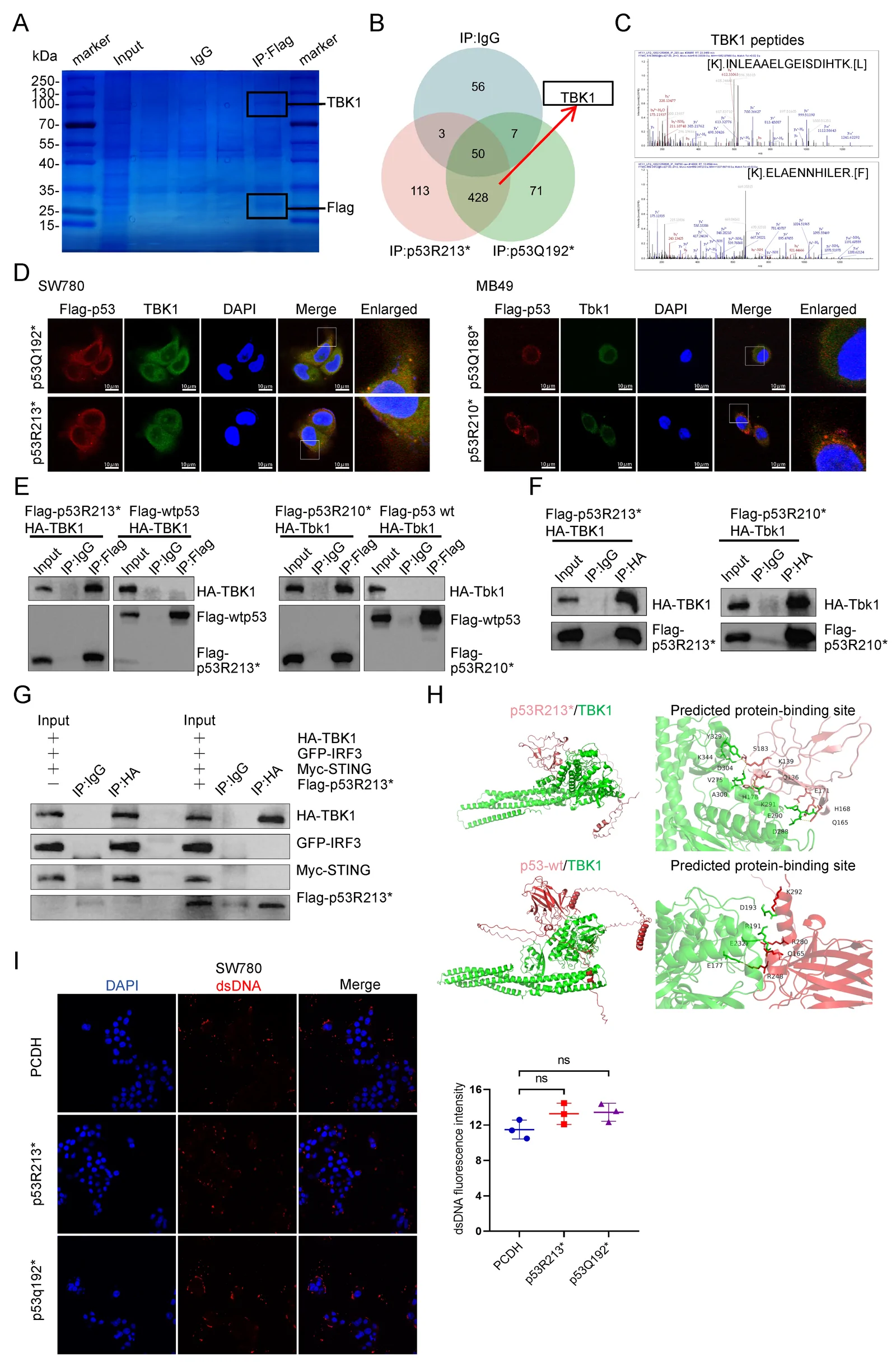
** E6-trunc p53 binds to TBK1 and prevents formation of the trimeric TBK1-STING-IRF3 complex.** (A) Immunoprecipitation of Flag-E6-trunc p53 from total cell lysates of E6-trunc p53 SW780 cells. Coomassie blue-stained protein electrophoresis gel showed the co-precipitated protein bands. (B) Mass spectrometry identification of co-precipitated proteins. The Venn diagram illustrates the inclusion of TBK1 among the co-precipitated proteins. (C) Mass spectrometry results displayed the characteristic peptide segments of TBK1. (D) Immunofluorescence showed the co-localization of E6-trunc p53 and TBK1 within cells. Scale bars, 10 μm. (E) Western blot analysis of cell lysates, immunoprecipitation of E6-trunc p53 and wtp53. (F) Immunoprecipitation of HA-TBK1 from total cell lysates of 293TN cells transfected with E6-trunc p53 and HA-TBK1. Immunoblot analysis of cell lysates and TBK1 immunoprecipitation. (G) 293TN cells were transfected with PCDH or p53R213*, and co-transfected with HA-TBK1, GFP-IRF3, and Myc-STING. Cell lysates were immunoprecipitated using HA antibody. Western blot analysis of cell lysates and immunoprecipitation. (H) Predicted structures of truncated and wild-type p53 and their putative TBK1-binding sites generated using AlphaFold-based structural modeling. (I) Cytosolic dsDNA immunofluorescence analysis in control and E6-trunc p53-expressing cells showed no significant difference in dsDNA signal intensity. Data were presented as mean ± SD. Statistical significance for panels I was determined using a two-tailed unpaired Student's t-test, ***p < 0.001.

**Figure 5:**
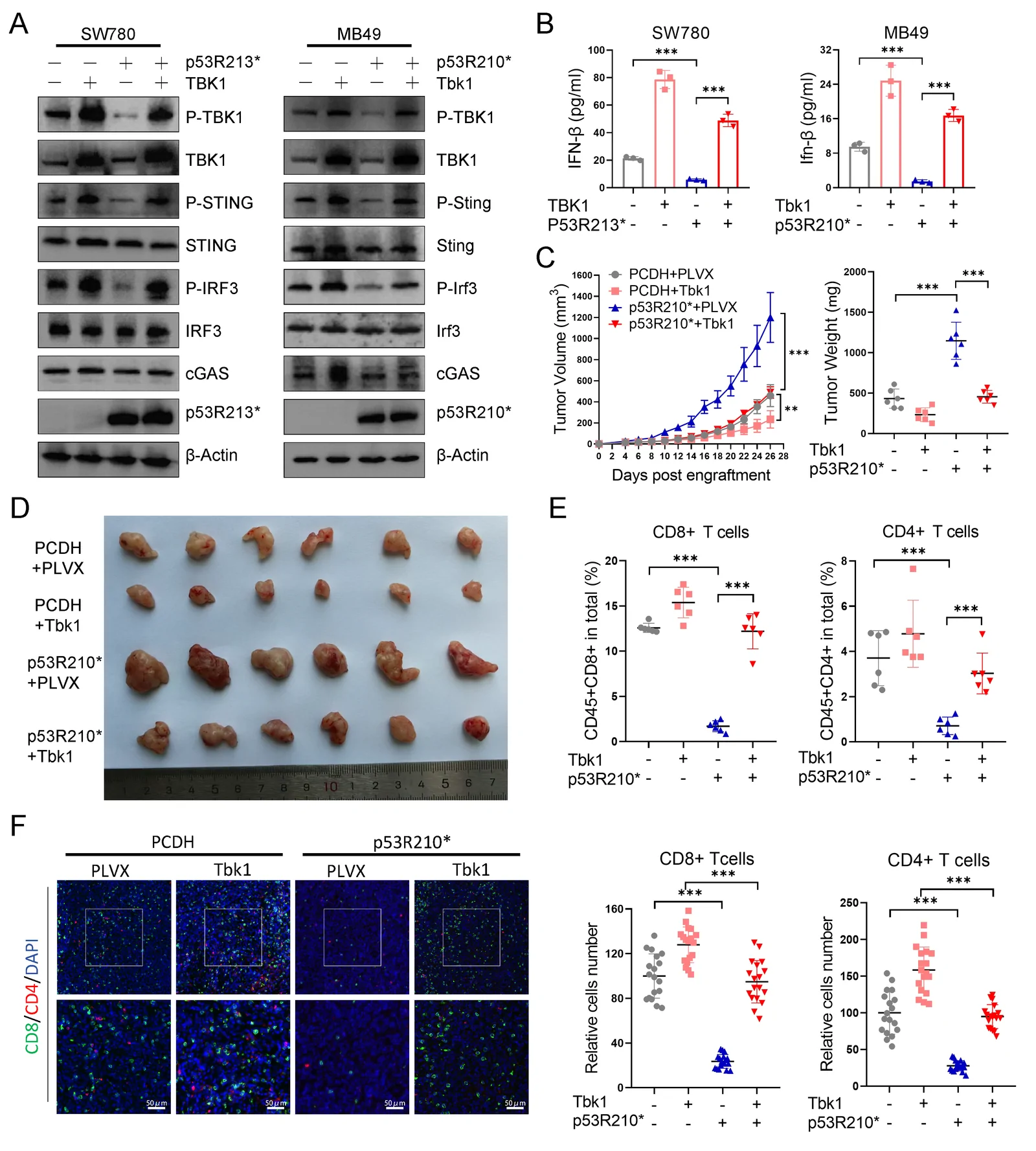
** Overexpression of TBK1 restores the type I IFN response suppressed by Exon-6-Truncated p53.** (A) Western blot analysis of SW780 and MB49 cells transfected with PCDH or E6-trunc p53 and re-transfected with PLVX or TBK1. (B) ELISA assay showed the expression levels of IFN-β protein in the indicated SW780 and MB49 cells. (C) Tumor growth curves and tumor weights in C57BL/6J mice (n = 6) subcutaneously injected with PCDH or E6-trunc p53 MB49 cells re-transfected with PLVX or TBK1. (D) Tumor images of C57BL/6J mice (n = 6) subcutaneously injected with the indicated MB49 cells. (E) Flow cytometric analysis of tumor-infiltrating CD8+ T cells and CD4+ T cells in the indicated MB49 tumors (n = 6). (F) Representative images and immunofluorescence quantification of CD8 and CD4 in the indicated MB49 tumors (n = 6). Scale bars, 50 μm. Data were presented as mean ± SD. Statistical significance for panels B, E, and F was determined using a two-tailed unpaired Student's t-test, while panel C utilized one-way ANOVA. **p < 0.01, ***p < 0.001.

**Figure 6:**
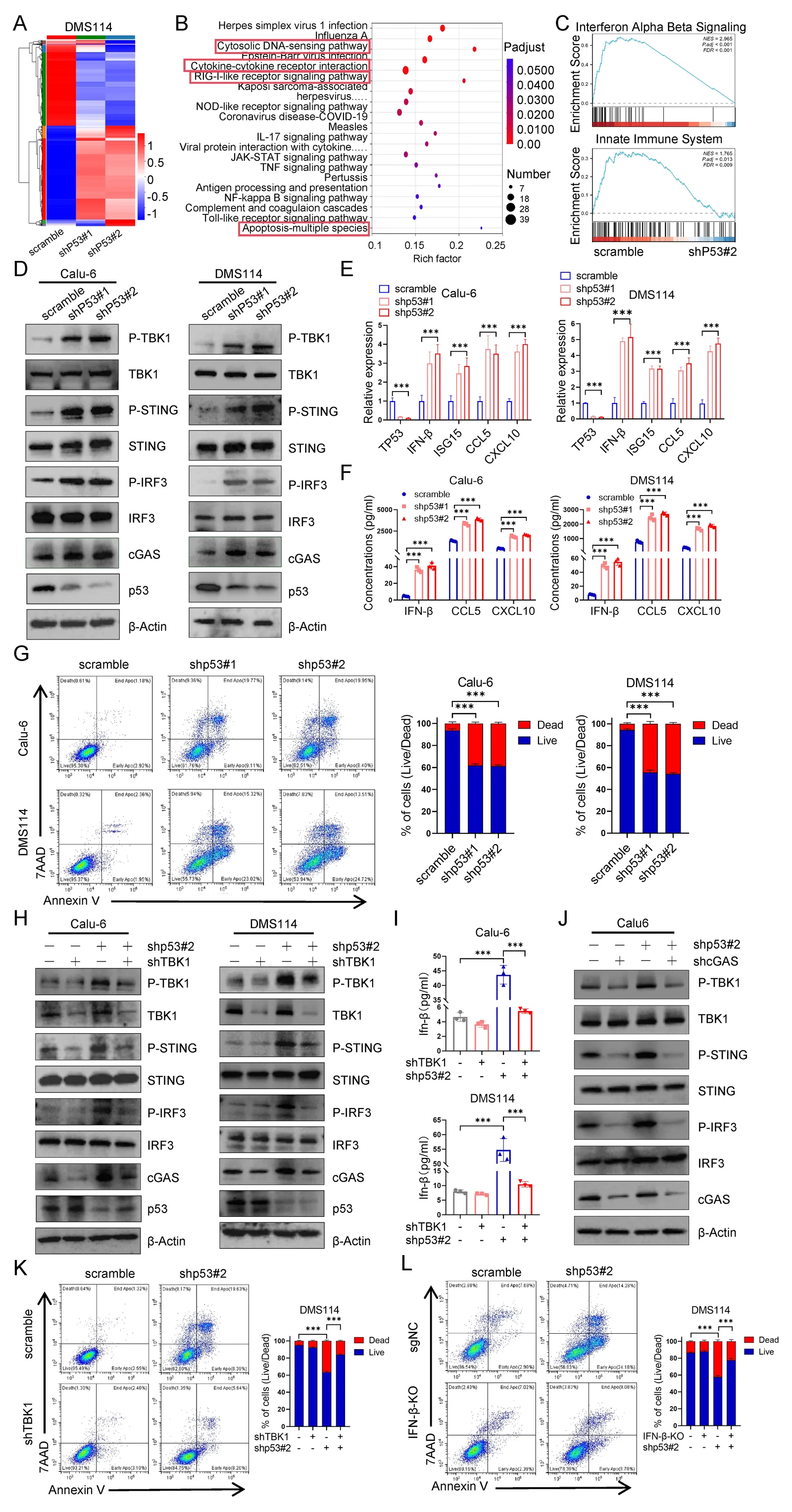
** Knockdown of E6-trunc p53 results in innate immune signaling activation and cell death.** (A) Unsupervised clustering heatmap depicted differentially expressed mRNAs in PLKO and shP53 DMS114 cells. (B) The top 20 significantly enriched KEGG pathways in shP53 DMS114 cells. (C) Representative GSEA plots of innate immunity-related gene sets in shP53 DMS114 cells. (D) Western blot analysis of PLKO and shP53 Calu-6 and DMS114 cells. (E) qRT-PCR analysis showed the expression levels of P53, IFN-β, ISG15, CCL5, and CXCL10 in the indicated Calu-6 and DMS114 cells. (F) ELISA assay showed the expression levels of IFN-β, CCL5, and CXCL10 proteins in the indicated Calu-6 and DMS114 cells. (G) Flow cytometric analysis of apoptosis in PLKO and shP53 Calu-6 and DMS114 cells. (H) Western blot analysis of PLKO or shTBK1 Calu-6 and DMS114 cells following P53 knockdown. (I) ELISA assay showed the expression levels of IFN-β protein in the indicated Calu-6 and DMS114 cells. (J) Western blot analysis of Calu-6 cells transfected with shP53 alone or co-transfected with shcGAS or shSTING. (K) Flow cytometric analysis of apoptosis in PLKO or shTBK1 Calu-6 and DMS114 cells following P53 knockdown. (L) Flow cytometric analysis of apoptosis in sgNC or IFNB-knockout Calu-6 and DMS114 cells following P53 knockdown. Data were presented as mean ± SD. Statistical significance for panels E, F, G, I, J, K and L was determined using a two-tailed unpaired Student's t-test. ns, not significant. ***p < 0.001.

**Figure 7:**
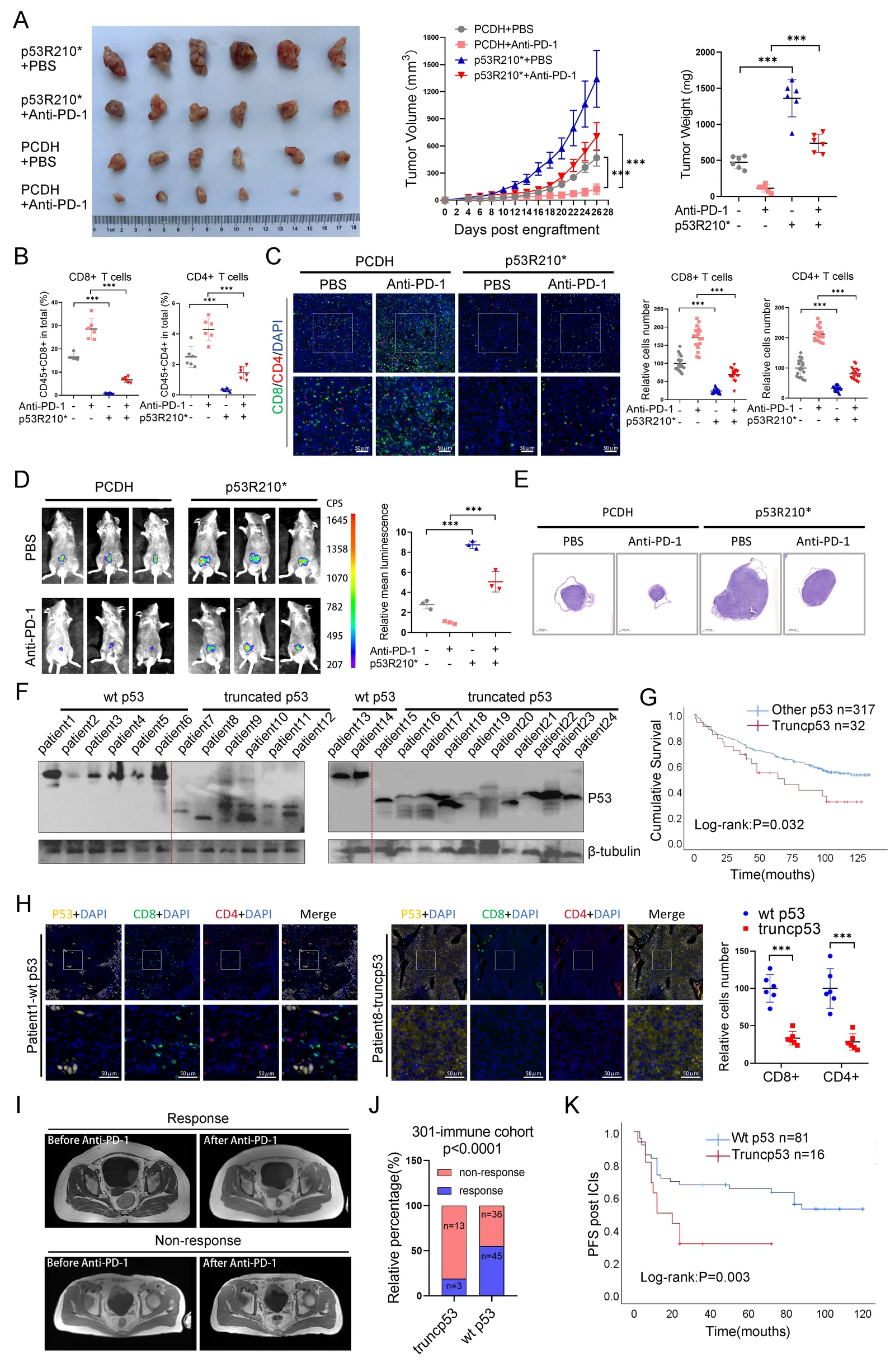
** Truncated p53 is associated with resistance to ICIs in bladder cancer.** (A) Tumor images, growth curves, and tumor weights of immunocompetent C57BL/6J mice (n = 6) subcutaneously injected with PCDH or E6-trunc p53 MB49 cells and treated with anti-PD-1 or control (PBS). (B) Flow cytometric analysis of tumor-infiltrating CD8+ T cells and CD4+ T cells in MB49 tumors (n = 6) from the indicated groups. (C) Representative images and immunofluorescence quantification of CD8 and CD4 in MB49 tumors (n = 6) from the indicated groups. Scale bars, 50 μm. (D) Representative bioluminescence images and histogram analysis of bioluminescence intensity in C57BL/6J mice (n = 6) with bladder orthotopic injection of luciferase-labeled PCDH or E6-trunc p53 MB49 cells, treated with anti-PD-1 or control (PBS). (E) Representative H&E staining of the indicated MB49 tumors. Scale bars, 2 mm. (F) Western blot showed the molecular weight of P53 protein in selected bladder cancer samples. (G) Kaplan-Meier analysis of cumulative survival in 349 bladder cancer patients with different TP53 genotypes. (H) Representative multiplex immunofluorescence images showed P53 (yellow), CD4 (red), CD8 (green), and DAPI (blue) in wild-type P53 and truncated p53 bladder cancer patients. Scale bars, 50 μm. (I) Representative MRI images of patients who responded to ICIs treatment versus non-responders at our center. (J) Comparison of immunotherapy efficacy between truncated p53 (n = 16) and wild-type P53 (n = 81) bladder cancer patients in the 301-immune cohort. (K) Progression-Free Survival (PFS) of truncated p53 patients (n = 16) versus wild-type P53 patients (n = 81) following ICI treatment. Data were presented as mean ± SD. Statistical significance for panels A, B, C, and D was determined using a two-tailed unpaired Student's t-test. Chi-square test was used for panel J. Survival analysis for panels G and K was performed using the log-rank test. ***p < 0.001.

## Data Availability

All data points in the figures are included in the supporting data file. The RNA-seq datasets supporting the conclusions of this article are available in the NCBI Gene Expression Omnibus (GEO) repository, [GSE308506, www.ncbi.nlm.nih.gov/geo/query/acc.cgi?acc=GSE308506].

## Abbreviations

E6-trunc p53: exon-6-truncated p53
NMIBC: non-muscle-invasive bladder cancer
MIBC: muscle-invasive bladder cancer
FGFR3: fibroblast growth factor receptor 3
IFN-I: type I interferon
IFN-β: interferon beta
PRRs: pattern recognition receptors
cGAS: cyclic GMP-AMP synthase
STING: stimulator of interferon genes
TBK1: TANK-binding kinase 1
IRF3: interferon regulatory factor 3
dsDNA: double-stranded DNA
RIG-I: retinoic acid-inducible gene I
MDA5: melanoma differentiation-associated protein 5
MAVS: mitochondrial antiviral-signaling protein
TLR: Toll-like receptor
LPS: lipopolysaccharide
ISGs: interferon-stimulated genes
NF-κB: nuclear factor kappa B
NK: natural killer
GZMB: granzyme B
PD-1: programmed cell death protein 1
PD-L1: programmed death-ligand 1
ICIs: immune checkpoint inhibitors
RNA-seq: RNA sequencing
GSEA: Gene Set Enrichment Analysis
KEGG: Kyoto Encyclopedia of Genes and Genomes
DEGs: differentially expressed genes
GEO: Gene Expression Omnibus
qRT-PCR: quantitative reverse transcription polymerase chain reaction
ELISA: enzyme-linked immunosorbent assay
Co-IP: co-immunoprecipitation
IHC: immunohistochemistry
H&E: hematoxylin and eosin
DAPI: 4′,6-diamidino-2-phenylindole
FFPE: formalin-fixed, paraffin-embedded
TUNEL: terminal deoxynucleotidyl transferase dUTP nick-end labeling
PBS: phosphate-buffered saline
SDS-PAGE: sodium dodecyl sulfate-polyacrylamide gel electrophoresis
PVDF: polyvinylidene difluoride
LC-MS/MS: liquid chromatography-tandem mass spectrometry
FDR: false discovery rate
RECIST: Response Evaluation Criteria in Solid Tumors
MRI: magnetic resonance imaging
PFS: progression-free survival
SD: standard deviation
ANOVA: analysis of variance
