# Supplementary material for: Exon-6 truncated p53 Suppresses Innate Immune Responses to Facilitate Immune Evasion in Bladder Cancer
Source: Int J Biol Sci. 2026 Sep 11;22(15):8251–70. doi: 10.7150/ijbs.131491 (PMC13685605; doi:10.7150/ijbs.131491)
Supplement: Supplementary file 1 [file ijbsv22p8251s1.pdf]

Fig.S1

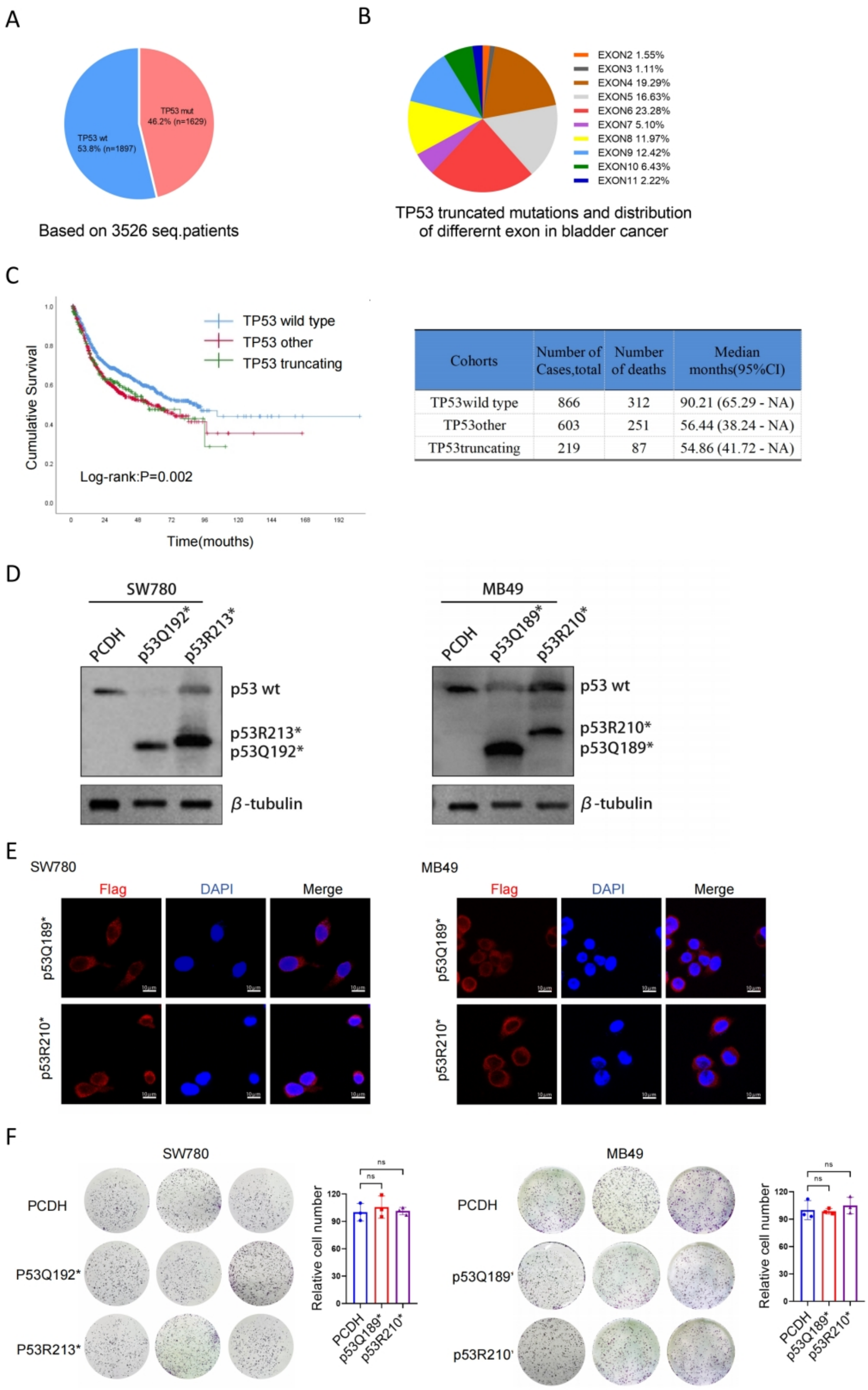

Figure S1. Patients with truncated TP53 mutations exhibit poor prognosis, and overexpression of E6-trunc p53 has no significant effect on in vitro cell proliferation.

(A) Proportion of TP53 gene mutations in bladder cancer samples from the TCGA, MSK, MDACC, BGI, and IGMBC databases.

(B) Proportion of TP53 gene mutation sites in exons across bladder cancer samples

from public databases.

(C) Kaplan-Meier analysis of cumulative survival in 1688 bladder cancer patients with different P53 genotypes from public databases.

(D) Western blot showed stable expression of E6-trunc p53 in SW780 and MB49 cells.To avoid band overexposure, the loading amount of overexpressed histones was reduced, resulting in lower levels of both endogenous and wild-type p53 proteins in the overexpression group compared with the control group.

(E) Immunofluorescence showed the predominant cytoplasmic localization of E6-trunc p53 in SW780 and MB49 cells.Scale bars, 10 μm.

(F) Colony formation assay showed no significant difference in the in vitro proliferation ability of the indicated cells.

Data were presented as mean ± SD. Survival analysis for panel C was performed using the log-rank test. Statistical significance for panel F was determined using a two-tailed unpaired Student's t-test. ns, not significant. ***$p < 0.001$.

Fig.S2

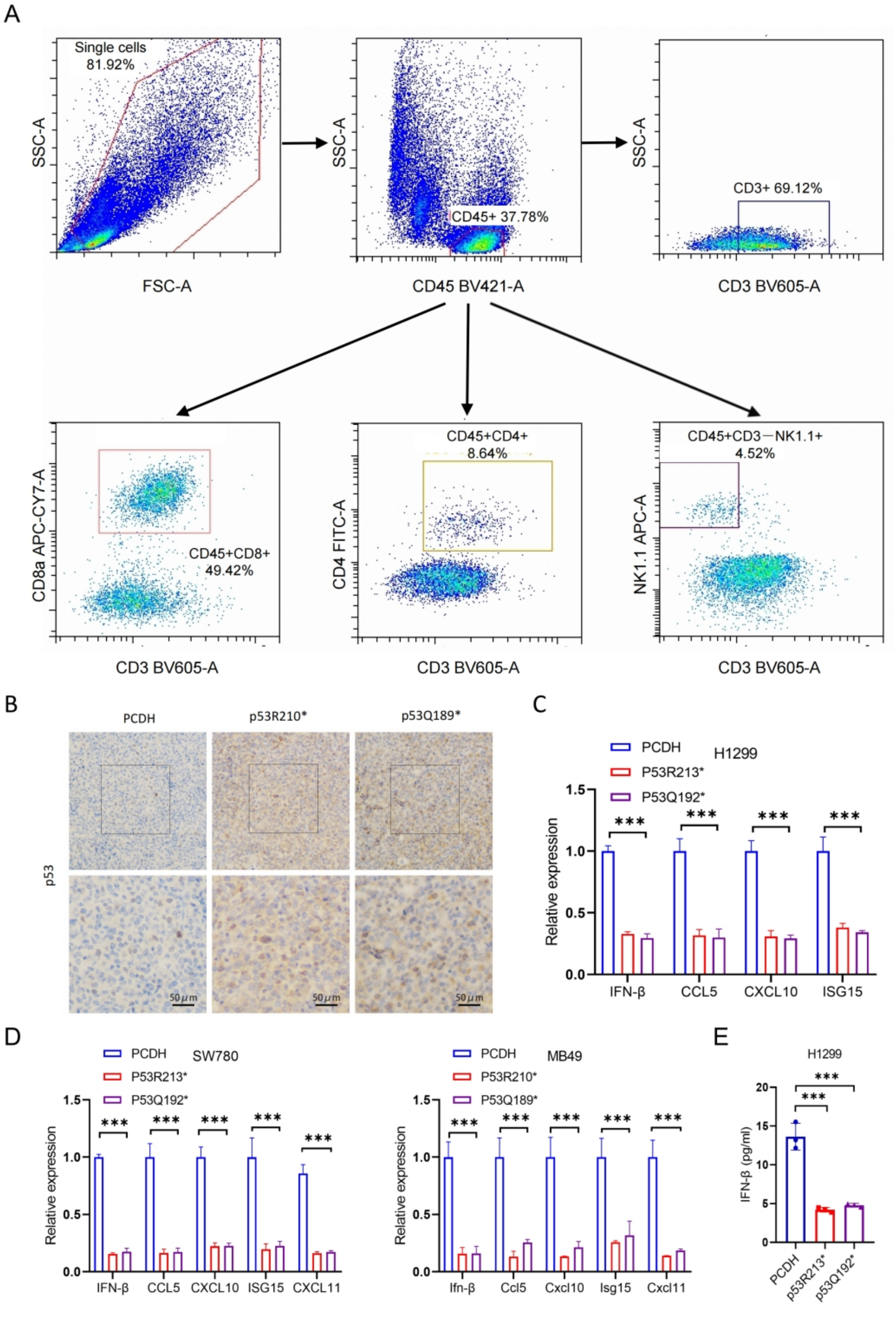

Figure S2. Representative flow cytometry gating strategy

(A) Representative flow cytometry gating strategy used to quantify the number of various immune effector cell subsets in the tumors of the indicated mice.

(B) Immunohistochemical staining of p53 in the indicated MB49 tumors (n = 6). Scale bars, 50 μm.

(C) qRT-PCR analysis of IFN-β, CCL5, CXCL10 and ISG15 mRNA expression in the indicated H1299 cells

(D) qRT-PCR analysis showed the expression levels of IFN-β, CCL5, CXCL10, ISG15, and CXCL11 in the indicated SW780 and MB49 cells.

(E) ELISA assay showed IFN-β protein levels in the indicated H1299 cells

Data were presented as mean ± SD. Statistical significance for panels C, D and E was determined using a two-tailed unpaired Student's t-test. ns, not significant. ***p < 0.001.

Fig.S3

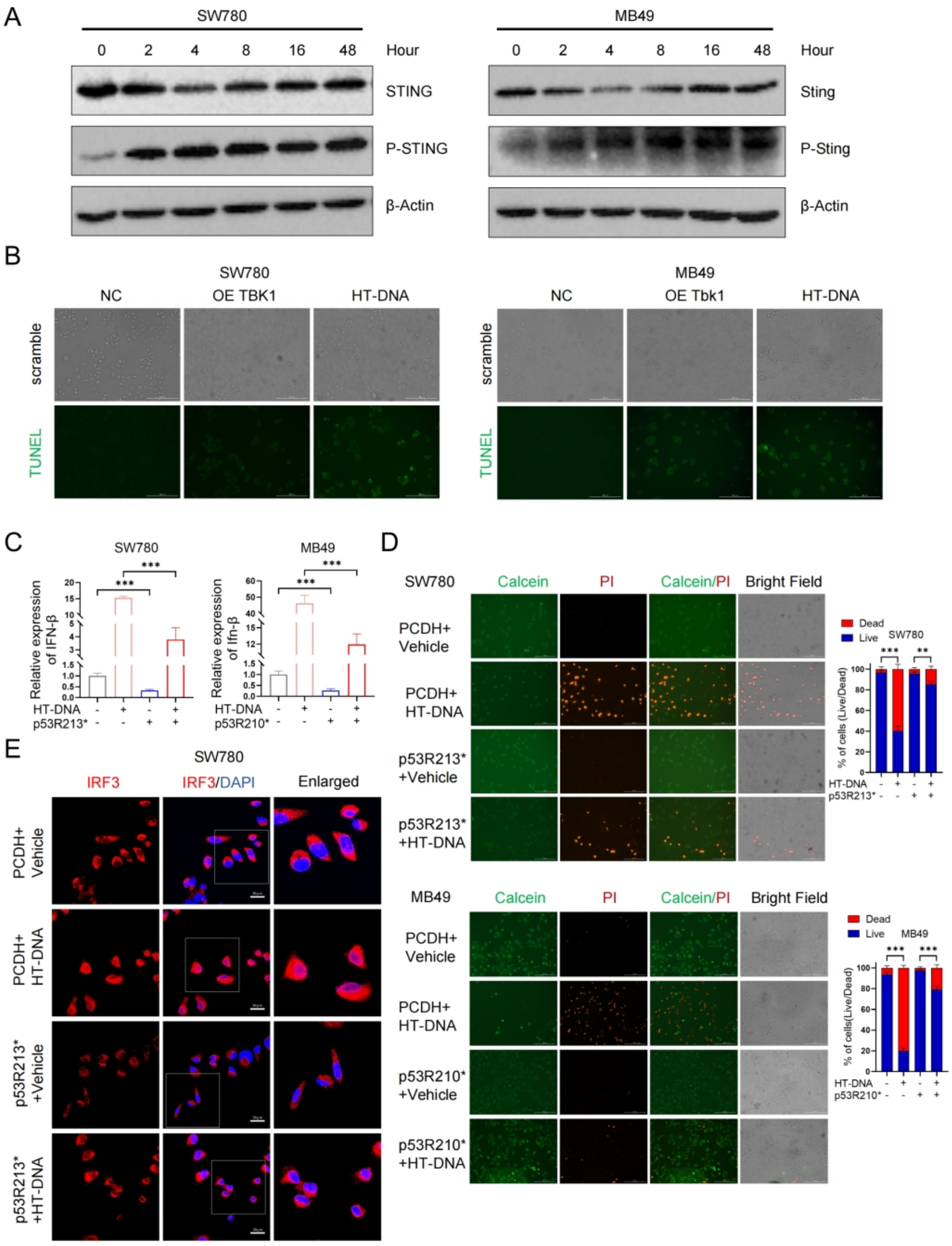

Figure S3.E6-trunc p53 suppresses innate immune signaling

(A) Representative Western blot showing time-dependent changes in total STING and phosphorylated STING following HT-DNA treatment.

(B) TUNEL staining of SW780 and MB49 cells transfected with TBK1, showing increased apoptotic cells relative to control.

(C) ELISA assay showed IFN-β protein levels in the indicatedSW780 and MB49 cells treated with HT-DNA.

(D) Calcein and PI staining showed the proportion of cell death in specified SW780 and MB49 cells treated with HT-DNA.

(E) Immunofluorescence staining of IRF3 in SW780 cells transfected with PCDH or E6-truncated p53, with or without HT-DNA treatment (4 μg/mL, 48 h). HT-DNA induced nuclear translocation of IRF3 in control cells, which was attenuated by E6-truncated p53 overexpression.

Data were presented as mean ± SD. Statistical significance for panels C and D was determined using a two-tailed unpaired Student's t-test. ns, not significant. ***$p < 0.001$.

Fig.S4

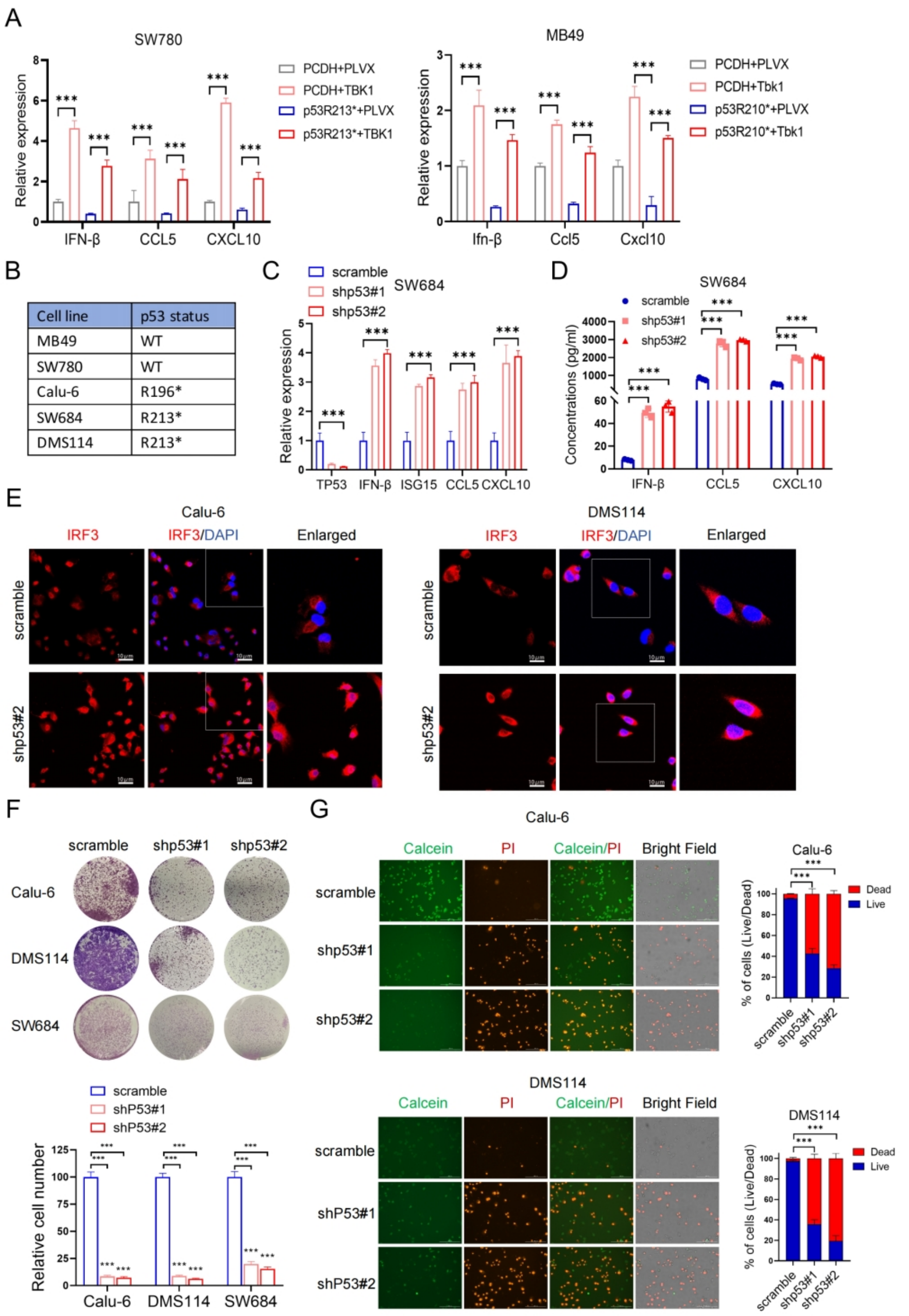

Figure S4. Upregulation of type I interferon and cell death following knockdown of E6-trunc p53.

(A) qRT-PCR analysis showed the expression levels of IFN-β, CCL5, CXCL10, ISG15, and CXCL11 in the indicated SW780 and MB49 cells.

(B) Table summarized the TP53 mutation status in the cell lines utilized in this study.

(C) qRT-PCR analysis showed the expression levels of P53, IFN-β, ISG15, CCL5, and CXCL10 in the indicated SW684 cells.

(D) ELISA assay showed the expression levels of IFN-β, CCL5, and CXCL10

proteins in the indicated SW684 cells.

(E) Immunofluorescence showed IRF3 nuclear translocation in shP53 Calu-6 and DMS114 cells.

(F) Crystal violet staining assay showed the proliferation of PLKO and shP53 Calu-6, DMS114, and SW684 cells.

(G) Calcein and PI staining showed the proportion of cell death in the indicated Calu-6 and DMS114 cells.

Data were presented as mean ± SD. Statistical significance for panels A, C, D, F, and G was determined using a two-tailed unpaired Student's t-test. ***$p < 0.001$.

Fig.S5

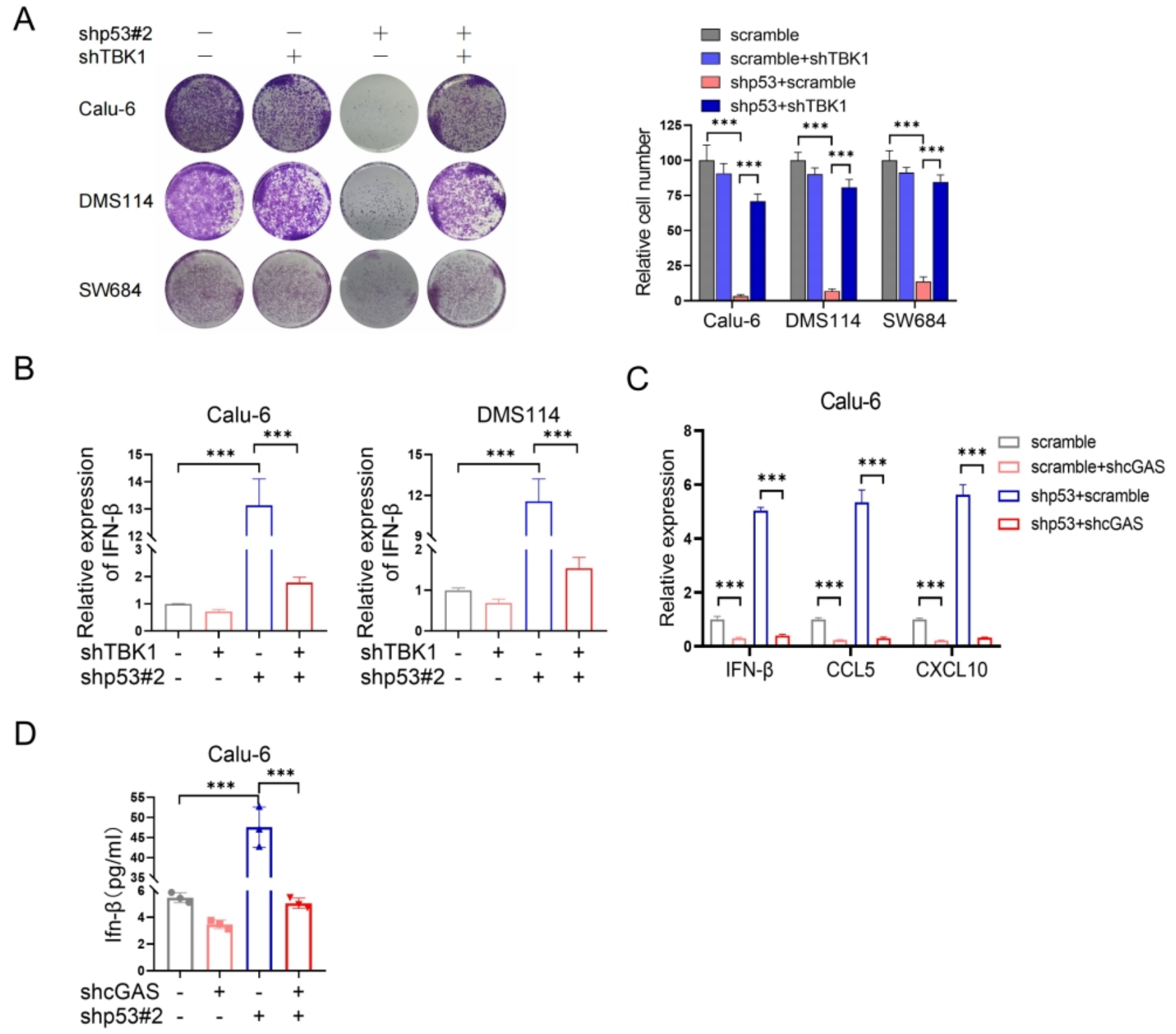

Figure S5. Knockdown of TBK1 or cGAS rescues cell death induced by E6-trunc p53 knockdown.

(A) Crystal violet staining assay showed the proliferation of the indicated Calu-6, DMS114, and SW684 cells.

(B) qRT-PCR analysis of IFN-β mRNA expression in the indicated Calu-6 and DMS114 cells.

(C) qRT-PCR analysis of IFN-β mRNA expression in Calu-6 cells transfected with shP53 alone or co-transfected with shcGAS or shSTING.

(D) ELISA assay showed IFN-β protein levels in the indicated Calu-6 cells transfected with shP53 alone or co-transfected with shcGAS or shSTING.

Fig.S6

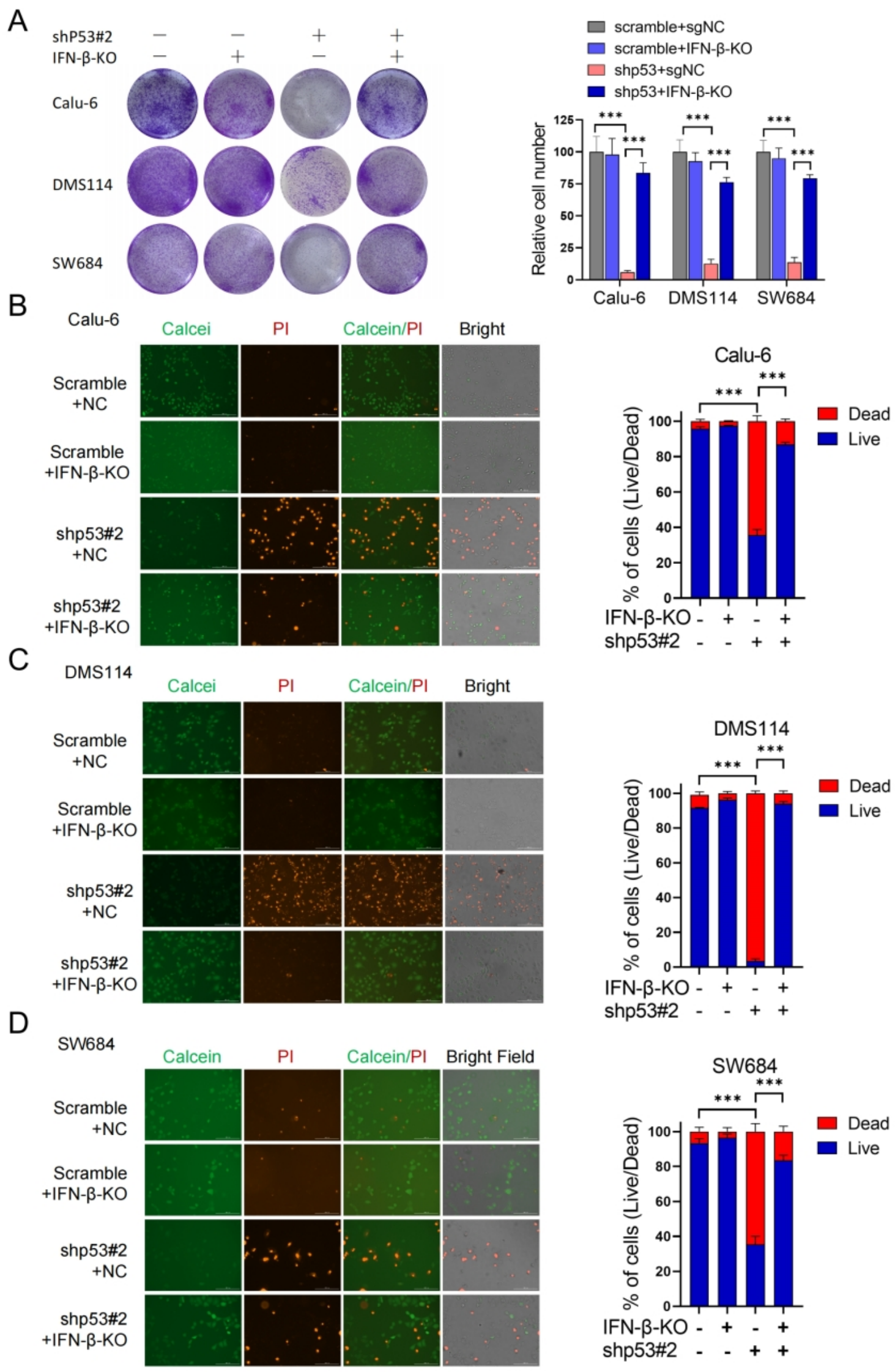

Figure S6. Knockout of IFN-β rescues cell death induced by E6-trunc p53 knockdown.

(A) Crystal violet staining assay showed the proliferation of the indicated Calu-6, DMS114, and SW684 cells.

(B) Calcein and PI staining showed the proportion of cell death in the indicated Calu-6 cells.

(C) Calcein and PI staining showed the proportion of cell death in the indicated DMS114 cells.

(D) Calcein and PI staining showed the proportion of cell death in the indicated SW684 cells.

Data were presented as mean ± SD. Statistical significance for panels A, B,C, and D was determined using a two-tailed unpaired Student's t-test. ***$p < 0.001$.

Fig.S7

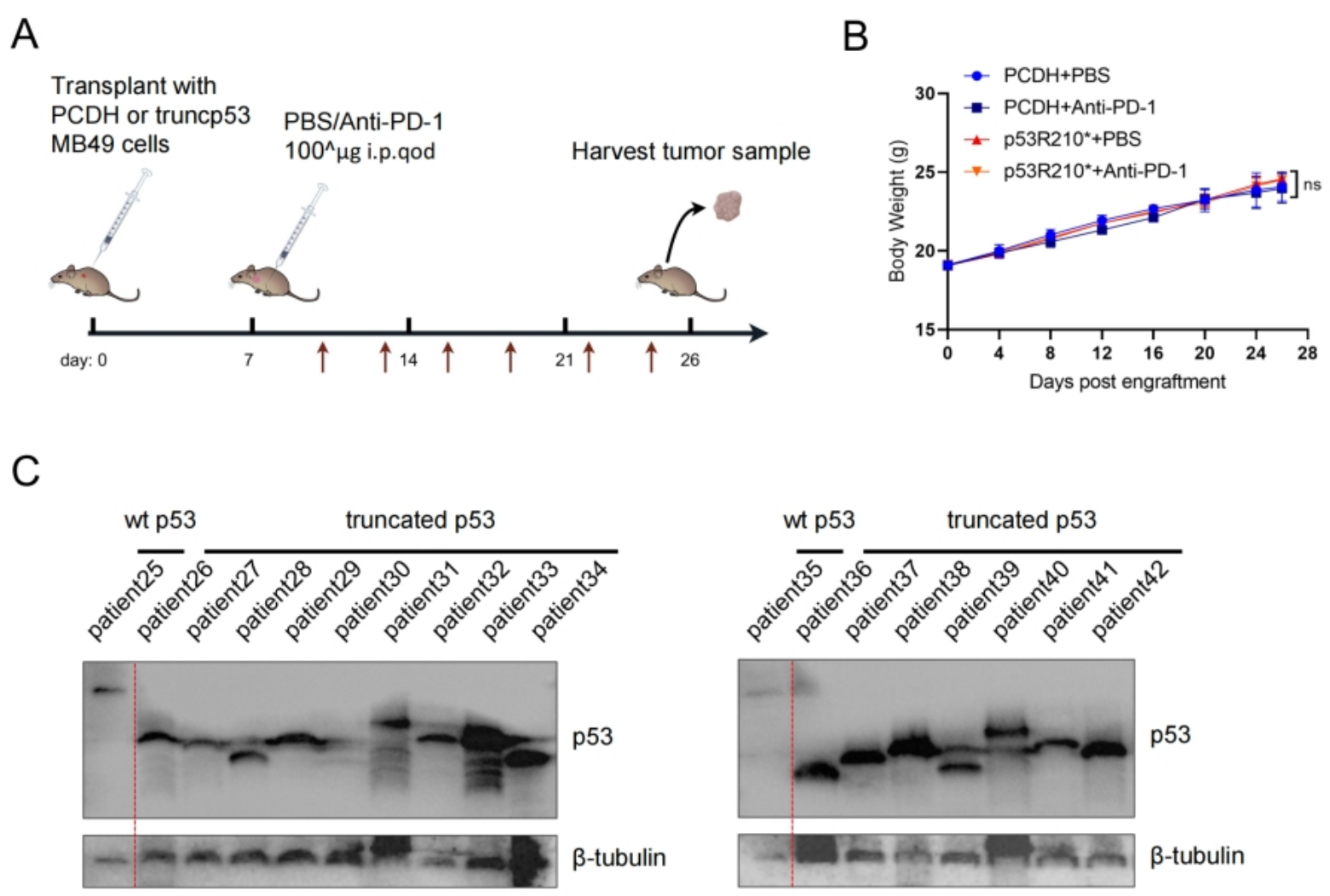

Figure S7. Safety validation of the immune therapy dose administered to mice and screening of truncated p53 bladder cancer samples.

(A) Body weight curve of the indicated mice (n = 6).

(B) Western blot showed the molecular weight of P53 protein in the screened bladder cancer samples.

(C) Western blot showed the molecular weight of P53 protein in selected bladder cancer samples.

Supplementaltable 1: The sequences of primers and oligonucleotides used in this study.	
**Primers for PCR（5’-3'）**	
**TP53 F**	CAGCACATGACGGAGGTTGT
**TP53 R**	TCATCCAAATACTCCACACGC
**IFN-β F**	ATGACCAACAAGTGTCTCCTCC
**IFN-β R**	GGAATCCAAGCAAGTTGTAGCTC
**CCL5 F**	CCATATTCCTCGGACACCACA
**CCL5 R**	TTCGGGTGACAAAGACGACTG
**CXCL10 F**	TGCCATTCTGATTTGCTGCC
**CXCL10 R**	TGCAGGTACAGCGTACAGTT
**ISG15 F**	CGCAGATCACCCAGAAGATCG
**ISG15 R**	TTCGTCGCATTTGTCCACCA
**CXCL11 F**	GACGCTGTCTTTGCATAGGC
**CXCL11 R**	GGATTTAGGCATCGTTGTCCTTT
**Trp53 F**	CACAGCACATGACGGAGGTC
**Trp53 R**	TCCTTCCACCCGGATAAGATG
**Ifn-β F**	CAGCTCCAAGAAAGGACGAAC
**Ifn-β R**	GGCAGTGTAACTCTTCTGCAT
**Ccl5 F**	ATATGGCTCGGACACCACT
**Ccl5 R**	CTTCGAGTGACAAACACGACTG
**Cxcl10 F**	TGCAAGTCTATCCTGTCCGC
**Cxcl10 R**	TCTTTGGCTCACCGCTTTCA
**Isg15 F**	CATCCTGGTGAGGAACGAAAGG
**Isg15 R**	CTCAGCCAGAACTGGTCTTCGT
**Cxcl11 F**	CCGAGTAACGGCTGCGACAAAG
**Cxcl11 R**	CCTGCATTATGAGGCGAGCTTG
**β-Actin F**	CACCATTGGCAATGAGCGGTTC
**β-Actin R**	AGGTCTTTGCGGATGTCCACGT
**β-actin F**	GGCTGTATTCCCCTCCATCG
**β-actin R**	CCAGTTGGTAACAATGCCATGT
**shRNA sequence**	
**shP53#1**	CCGGTCAGACCTATGGAAACTACTTCTCGAGAAGTAGTTTCCATAGGTCTGA
**shP53#2**	CCGGGTCCAGATGAAGCTCCCAGAAttcaagagaTTCTGGGAGCTTCATCTGGAC
**shTBK1**	GATCCGAGAGCACTTCCAACCATCTTTCAAGAGAAGATGGTTGGAAGTGCTCTTTTTTGGAAA
**sgRNA sequence**	
**sgIfn-β#1**	TCCATGAGCTACAACTTGCT
**sgIfn-β#2**	GCCTCCCATTCAATTGCCAC
**sgIfn-β#3**	GACTATTGTTGAGAACCTCC
